# In Situ Surface-Sensitive Investigation of Multiple Carbon Phases on Fe(110) in the Fischer–Tropsch Synthesis

**DOI:** 10.1021/acscatal.2c00905

**Published:** 2022-06-13

**Authors:** Mikhail Shipilin, David Degerman, Patrick Lömker, Christopher M. Goodwin, Gabriel L. S. Rodrigues, Michael Wagstaffe, Jörgen Gladh, Hsin-Yi Wang, Andreas Stierle, Christoph Schlueter, Lars G. M. Pettersson, Anders Nilsson, Peter Amann

**Affiliations:** † Department of Physics, Stockholm University, 10691 Stockholm, Sweden; ‡ Photon Science, Deutsches Elektronen-Synchrotron DESY, 22607 Hamburg, Germany; § DESY NanoLab, Deutsches Elektronen-Synchrotron DESY, 22607 Hamburg, Germany; ∥ PULSE Institute, SLAC National Accelerator Laboratory, Menlo Park, 94305 California, United States; ⊥ Physics Department, University of Hamburg, 20148 Hamburg, Germany

**Keywords:** Fischer−Tropsch, iron carbide, hydrogenation, carburization, heterogeneous catalysis

## Abstract

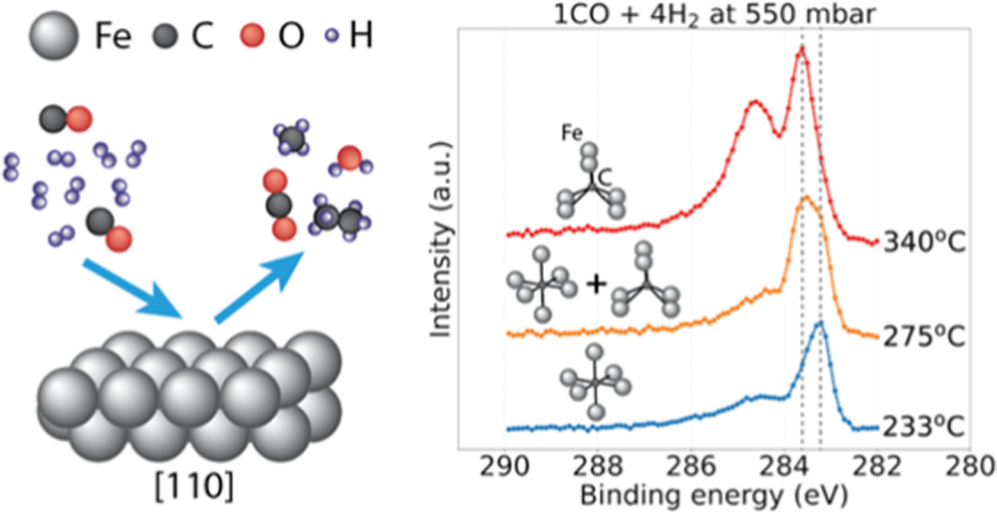

Carbide formation on iron-based catalysts is an integral and, arguably, the most important part of the Fischer–Tropsch synthesis process, converting CO and H_2_ into synthetic fuels and numerous valuable chemicals. Here, we report an in situ surface-sensitive study of the effect of pressure, temperature, time, and gas feed composition on the growth dynamics of two distinct iron–carbon phases with the octahedral and trigonal prismatic coordination of carbon sites on an Fe(110) single crystal acting as a model catalyst. Using a combination of state-of-the-art X-ray photoelectron spectroscopy at an unprecedentedly high pressure, high-energy surface X-ray diffraction, mass spectrometry, and theoretical calculations, we reveal the details of iron surface carburization and product formation under semirealistic conditions. We provide a detailed insight into the state of the catalyst’s surface in relation to the reaction.

## Introduction

Iron is ubiquitously used as a base catalyst material for Fischer–Tropsch synthesis (FTS)—the process of converting a mixture of CO and H_2_ into various hydrocarbon (HC) molecules necessary for the production of synthetic fuels, waxes, and numerous other chemicals.^1^ The benefit of iron-based catalysts is defined by the low price of the active material, high tolerance to sulfur poisoning, relatively low methane selectivity, and the propensity of iron to catalyze the water gas shift (WGS) reaction.^2^ The latter, in particular, allows for the conversion of hydrogen-lean or CO_2_-rich gas mixtures, which is preferable for the conversion of sustainable feedstock, such as the products of biomass gasification.^3,4^

A large number of studies of iron-based FT catalysts show that several iron carbide (e.g., ε-Fe_2_C, ε′-Fe_2.2_C, Fe_7_C_3_, χ-Fe_5_C_2_, Θ-Fe_3_C) and (hydr)oxide (α,γ-Fe_2_O_3_, Fe_3_O_4_, FeO, FeOOH) phases, as well as metallic iron and elemental carbon in various forms, can be present in different combinations during the conversion process.^5−9^ This complexity has caused an extensive debate surrounding the catalyst-active phase, reaction and deactivation mechanisms, selectivity issues, effect of the support, and ultimately potential ways to design novel improved catalysts.^7,10^ The recently prevailing opinion favors iron carbide phases as promoting carbon chain growth and thus governing the FT activity in general.^9−12^ Regardless of whether it is the only mechanism, or merely one of the multitudes of possible reaction pathways, surface carburization seems to be an integral part of iron-based FTS, making a detailed understanding of the carburization process and its dependence on the reaction conditions of paramount importance.

Although the amount of literature discussing iron carbides appearing during FTS is vast, surface-sensitive in situ studies of carbide formation under elevated pressure on model iron catalysts are scarce.^13^ The difficulty in obtaining systematic and conclusive results on this system is defined by the presence of many variable parameters in the experiment, such as temperature, pressure, gas feed ratio, type of catalyst, catalyst composition, promoters, and type of reactor. In addition, the harsh conditions (∼250–350 °C temperature and ∼10–20 bar pressure) of the FTS process present a further challenge for experimental studies due to the limited applicability of most surface-sensitive techniques to in situ measurements of the chemical and structural composition of the working catalyst under operating conditions. Consequently, existing studies are dominated by theoretical approaches, experiments performed under vacuum conditions, ex situ studies of samples that have been exposed to FT conditions, and non-surface-sensitive measurements averaging over the bulk of the catalyst. The possibility to apply highly sensitive surface science techniques to well-defined model systems under the conditions of the ongoing FTS process would undoubtedly improve the understanding of the reaction mechanism and help to promote the design of efficient FT catalysts.

X-ray photoelectron spectroscopy (XPS) is a highly established technique within the field of surface studies in catalysis. The energy distribution of the core photoelectrons escaping from the sample upon ionization using X-rays provides detailed insight into the chemical state and transformations of species located in the surface region.^14,15^ However, under nonvacuum conditions, the short inelastic mean free path (IMFP) of the escaping photoelectrons has hitherto limited the possibilities when applying XPS since the gas molecules prevent the electrons from reaching the detector. Successful studies of catalyst surfaces have been conducted using differentially pumped XPS systems that operate at pressures around 1 mbar.^16^ However, hydrogenation reactions, such as FT, require higher pressures to reach significant reactant turn-over rates. The POLARIS instrument at the P22 beamline of the PETRA III synchrotron at DESY employed in this work was specifically designed for investigating catalytic hydrogenation processes under significantly more elevated pressures.^17,18^

Surface X-ray diffraction (SXRD) is a photon-in–photon-out experimental technique that can operate under elevated pressures and delivers surface-specific information about the ordered structures and structural transformations on the surface.^19−21^ It is an ideal complement to XPS since the latter focuses on the electronic and chemical states of the species while lacking the ability to determine the surface morphology.

**Figure 1:**
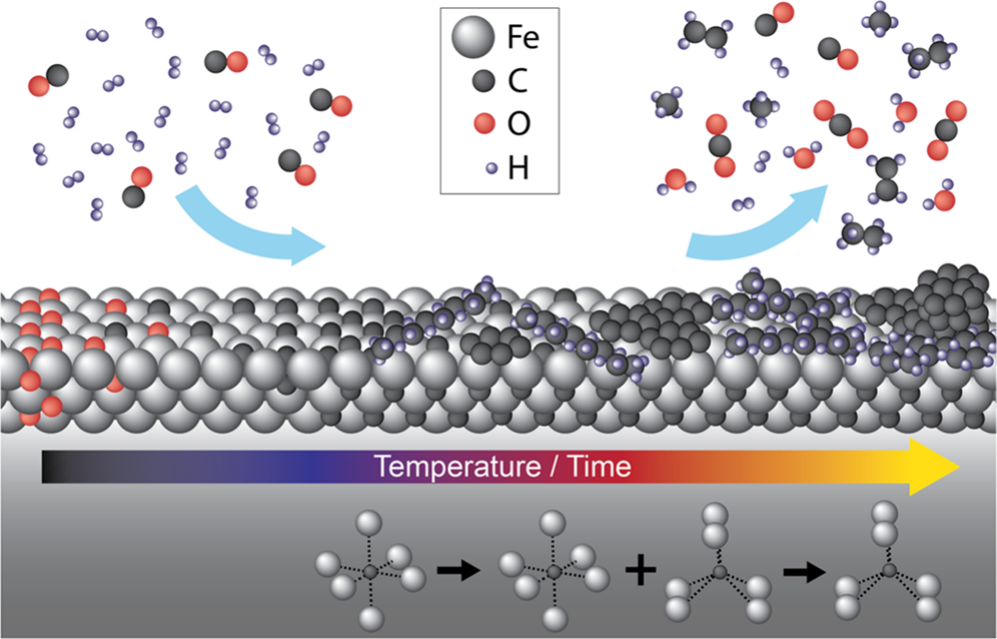
Generalized schematic drawing of the surface evolution process observed in situ and reported in the current contribution. An Fe(110) single-crystal surface exposed to CO hydrogenation conditions sequentially undergoes the process of oxide reduction, two-step carburization, and the formation of long-chain hydrocarbon molecules, graphite, and coke.

Here, we report in situ spectroscopic observations of the chemical evolution of an Fe(110) single-crystal surface acting as a catalyst in the FT process under unprecedented gas pressure of up to 700 mbar, which is orders of magnitude higher than any other photoelectron spectroscopy measurements previously performed for similar systems.^22−25^ These investigations are complemented by diffraction studies of the same system under similar conditions. For all gas compositions and pressures in the XPS experiment, adsorbed oxygen atoms and iron oxide phases present in the lower-temperature range (close to 150 °C) gradually disappear with increasing temperature. Simultaneously, the formation of atomic carbon and subsequent carburization of the surface region takes place in a two-step process, developing with both temperature and time. With support from theoretical calculations, two types of carbon atoms surrounded by Fe in octahedral (O) and trigonal prismatic (ΤP) geometries were identified to form on the surface, completely converting the iron metal to iron carbide compounds within the probed depth of a few tens of atomic layers. Their growth dynamics are strongly dependent on the pressure and gas composition. More species, likely including long-chain HCs, as well as sp^2^-hybridized (graphitic) carbon and coke characteristics for FTS, also appear on the surface to a varying extent depending on the reaction conditions. At the same time, a perceptible increase in temperature in the methyl signal (mass 15 amu) detected by mass spectrometry indicates the catalytic activity of the surface in the experiment. These findings extend the knowledge of the FTS process with a unique in situ insight into the initial stages of pressure-, time-, temperature-, and gas-composition-dependent iron carburization and FTS reaction mechanism. A generalized schematic drawing of the observed surface evolution is shown in Figure 1.

## Materials and Methods

### Experimental Setups

For the details on XPS and diffraction experimental setups and mass spectrometry implementation, see Section S1 in the Supporting Information.

### Samples and Gases

In all experiments, the surface of a 4N5 purity Fe(110) single crystal purchased from surface preparation lab (SPL) was prepared by multiple cycles of 30 min Ar^+^ ion sputtering at 1.5 kV and annealing at up to 700 °C for 5 min, alternating with hydrogen treatment at a 10–100 mbar pressure and a 400 °C temperature (occasionally, the H_2_ flow was exchanged with the CO_2_ flow for mild surface oxidation) until the survey photoelectron spectra confirmed the absence of contamination of the surface. In diffraction experiments, the detected pattern was required to represent a flat metallic surface. Representative survey spectra and diffraction patterns of the surface before and after a set of measurements can be found in the Supporting Information (Figures S2-1,2,3 and S7-1,2,3). Mild oxidation of the surface in the form of iron oxide was not considered as a contamination since it is notoriously difficult to keep iron metallic outside ultrahigh vacuum (UHV) conditions and it inevitably oxidizes upon dozing of the reaction gas mixture and reduces later in the carburization process. The control experiments (see Figure S3-1,2 in the Supporting Information) with and without the photon beam show no principal difference, allowing us to assume that there were no significant beam-induced effects that would drastically change the observed surface behavior.

The clean sample surface was exposed to gas mixtures with different relative concentrations of 1:1 and 1:10 for CO and H_2_, respectively. Both gases were supplied from 5N purity bottles. In the photoelectron spectroscopy experiments, the gases were additionally filtered in-line prior to entering the gas mixing system by Maxi Gaskleen and MicroTorr MC45-904F gas purifiers for CO and H_2_, respectively. In the diffraction experiments, the CO was filtered of potential carbonyl contamination prior to entering the reaction chamber by an LPM Carbonyl Trap CT2.0. The gas flows were controlled by Bronkhorst mass flow controllers, and the total flow within the same experiment was kept constant for different gas mixtures.

The temperature was measured by an N-type thermocouple placed between the heater and the sample’s backside. The temperature values were corrected using calibration measurements, correcting the difference between the front and backsides of the crystal (see Section S4 of the Supporting Information for further details), which is important due to the cooling effect of the gas at high pressure and flow.

### XPS Data Processing

All XPS data shown in the current contribution have undergone the following corrections. First of all, the binding energy (BE) in the spectra was referenced to the Fermi level (see Figure S5-1 in the Supporting Information for an example), which was measured after every change of conditions and stayed constant throughout the measurements. Second, the BE was corrected by the recoil effect (see Section S1 in the Supporting Information for more details) known to shift the apparent BE of light adsorbates toward higher values due to the partial transfer of kinetic energy of the escaping electron to the emitting atom when the excitation is induced by high-energy photons.^26,27^ For C 1s, O 1s, and Fe 2p spectral lines, the values of the shift are 0.210, 0.158, and 0.045 eV, while the broadening is roughly 0.099, 0.086, and 0.046 eV at room temperature (and 0.144, 0.125, and 0.067 eV at 300 °C), respectively. Although small, these corrections are important when discussing various hydrocarbon and carbon species on the surface since they are often very close to each other in BE. It should be noted that since all spectra are referenced to the Fermi level of iron, they are implicitly shifted by 0.045 eV before applying the recoil effect corrections.

Further, the spectra were normalized by the number of sweeps and by the dwell time, transforming the recorded signal into universal counts per time unit. Then, the spectra within one set of C 1s, O 1s, and Fe 2p recorded simultaneously at a single temperature and pressure were normalized to the background level around 282 eV in the C 1s spectrum, which is the lowest recorded BE of the set. The remaining constant background was removed, while other types of background were left intact and were later included in the fitting procedure. Lastly, the spectral intensity was corrected by the value of the corresponding photoionization cross section^28,29^ and the corresponding coefficient for photoelectron total scattering in gas given by
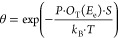
where *P* is the gas pressure between the sample and the analyzer nozzle, *O*_T_(*E*_e_) is the total gas-phase scattering cross section of the photoelectrons with the kinetic energy *E*_e_ (for gas mixtures, the weighted mean of the scattering cross sections was taken), *S* is the distance between the sample surface and the vacuum part of the analyzer, *k*_B_ is the Boltzmann constant, and *T* is the temperature. The temperature of the gas was assumed to be close to room temperature.

The fitting of spectra was performed using algorithms written in Python analogous to generally accepted approaches realized in, e.g., CasaXPS software. The code is publicly available on GitHub and PyPI.^30,31^ The XPS peaks were most often fitted using Pseudo Voigt profiles^32^—convolution of the Lorentzian (describing the core-hole lifetime effect) with the Gaussian containing the temperature-dependent phonon broadening and the instrumental function. For species that represent electric conductors, the Doniach–Šunjić (DS) line shape^33^ was used and demonstrated a good fit. The criteria for fitting were the following: a specific component was added only if it was clearly present in at least one spectrum of a compared set. The resulting fitted BE position and Gaussian/Lorentzian contribution of each peak were forced to be the same for the whole compared set of spectra. A Shirley-type function was used for background correction.

### Mass Spectrometry

The mass spectrometry signal was recorded for a number of masses, *m*/*z* = 15, 16, 18, 28, and 44 a.u. Mass 28 a.u. corresponds to CO gas and was used for normalization of other signals to enhance their visibility. The signals were also normalized by the dwell time. The signal at mass 15 a.u. corresponding to the CH_3_ radical was chosen as the main indicator of the hydrogenation reaction activity.

### Computational Details

Density-functional theory (DFT) calculations were performed with the Vienna Ab initio Simulation Package (VASP5)^34^ using the Perdew–Burke–Ernzerhof (PBE)^35^ exchange–correlation functional in conjunction with projector augmented-wave (PAW) potentials,^36^ a plane-wave energy cutoff of 520 eV, and a Monkhorst–Pack *k*-point sampling density of 2.5 *k*-points/Å^3^ in the supercell. Initial bulk structures for analyzed iron carbides were taken as POSCAR files from the Materials Project database^37^ with the exception of ε-Fe_2_C, which structural parameters were taken from the literature^38^ and the structure was built using the atomic simulation environment (ASE).^39^

The XPS BE shifts were obtained in two different independent manners: (i) simulating a core-ionized state, where a core hole is explicitly calculated in the final state, and (ii) making use of the core equivalent or Z + 1 approximation.^40,41^ In both cases, the BE is obtained using the equation
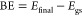
where *E*_final_ is the total electronic energy of the final state, and *E*_gs_ is the total electronic energy of the initial or ground state.

In all cases, the number of calculated bands were chosen as *n*_e/2_ + 150, where *n*_e_ is the total number of electrons in the supercell.

## Results and Discussion

### XPS Experiment

**Figure 2:**
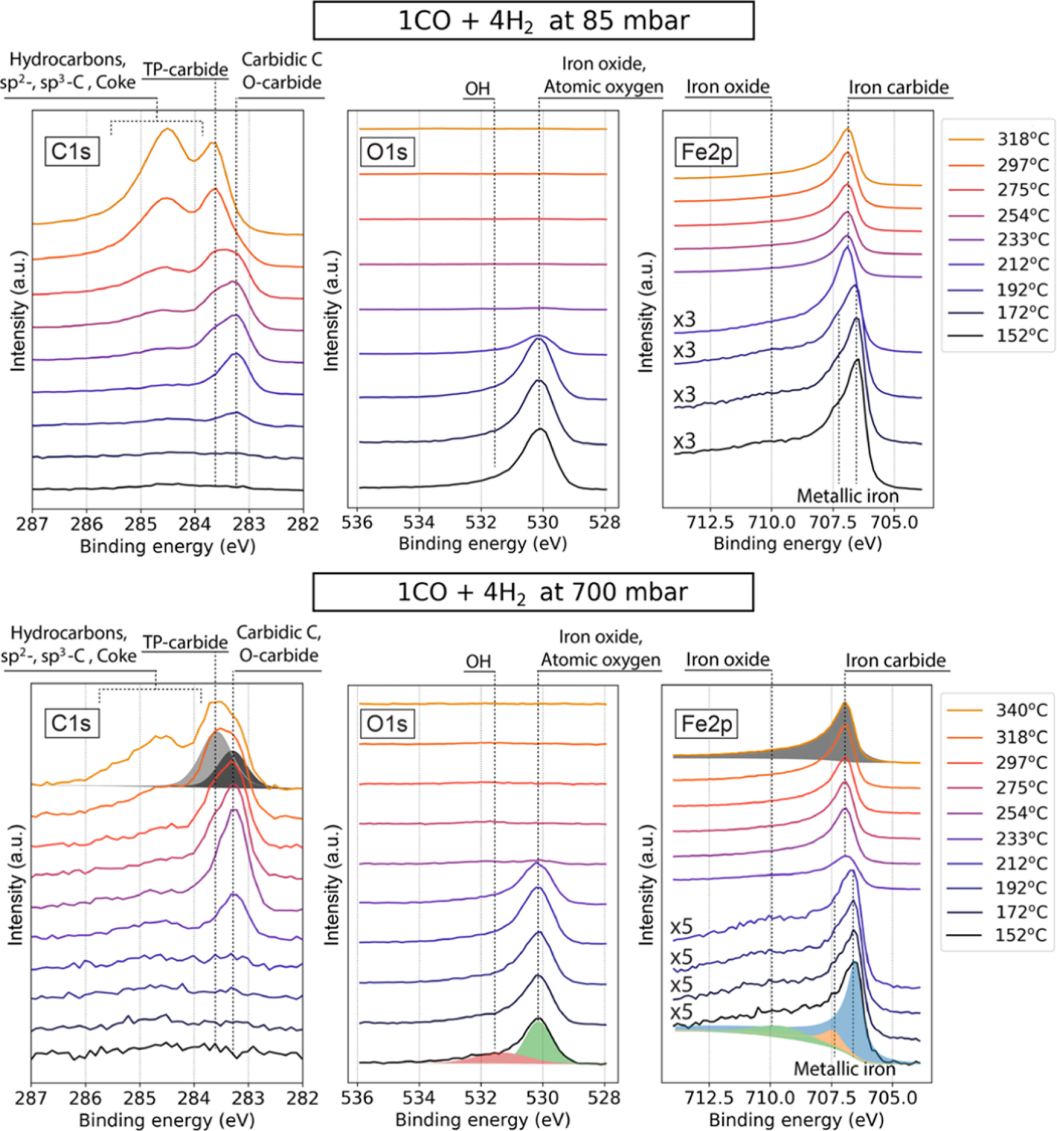
Photoelectron spectra of C 1s, O 1s, and Fe 2p_3/2_ regions recorded using 4.6 keV photons at 85 (top row) and 700 (bottom row) mbar in the 1CO:4H_2_ gas mixture at a 2 L*_n_*/min (L*_n_* is the gas volume in liters at atmospheric pressure) total flow. Note that the lower-temperature spectra of the Fe 2p_3/2_ region are magnified by factors of 3 and 5 for improved visibility of the oxide contribution. Approximate locations of determined species are annotated in the figure. “TP-” and “O-carbide” stand for carbide structures with trigonal prismatic and octahedral sites occupied by carbon atoms, respectively. Examples of spectra fitting are shown for selected measurements at 700 mbar.

In the current work, an Fe(110) single-crystal surface was prepared in ultrahigh vacuum (UHV) until no surface contaminants were seen in the photoelectron survey spectra (see the Materials and Methodsand Section S2 in the Supporting Information for details). After preparation, the crystal was exposed to reaction gas mixtures of CO and H_2_ at 1:1, 1:2, 1:4, and 1:10 ratios, 150 °C temperature, and pressures in the range from 85 to 700 mbar. The temperature was then increased stepwise. At each temperature step, sets of XP spectra of the C 1s, O 1s, and Fe 2p regions were acquired. Such sets recorded at 85 and 700 mbar in the 1CO:4H_2_ gas mixture are shown in Figure 2. These spectra are representative of all examined cases since the same species are observed for other reaction conditions. Important differences between the sets will be discussed further.

The surface becomes slightly oxidized at low temperature when the reaction mixture is introduced to the sample surface, which is indicated by the O 1s signal around 530.1 eV binding energy (BE) and the Fe 2p_3/2_ signal around 710.0 eV.^42^ The latter is broad and weak and is dominated by the Fe^0^ multiplet signal with components at 706.5 and 707.4 eV.^43,44^ Since the iron oxide contribution of around 710.0 eV is small in comparison with the Fe^0^ signal, one can conclude that the oxygen-containing phase is scarce. The C 1s spectra at low temperature are quite weak, with a minuscule presence of the chemisorbed carbidic carbon signal at 283.3 eV and the adventitious sp^2^ carbon around 284.6 eV.^45^

Upon increase of the temperature, the signal at 530.1 eV and around 710.0 eV is reduced, while the Fe^0^ multiplet turns into a single component at approximately 706.9 eV, indicating carburization of iron.^5,46−48^ The C 1s region at the same time develops, at first, a peak at 283.3 eV, followed by an increase of the signal at 283.6 eV and a broad feature spanning the interval between approximately 283.8 and 285.8 eV.

The signal from the gas phase was always present in both C 1s and O 1s spectra at around 294 eV and 538 eV, respectively. To achieve a higher time resolution of the experiment, it was not measured.

It should be noted that at the chosen experimental settings of a 4600 eV photon energy and a 0.4° incident angle, which is below the critical angle of the total external reflection for iron at that energy providing the maximum surface sensitivity, the attenuation length of the X-ray wave is only 2.26 nm in iron and about 3.18 nm in iron oxide, as calculated using Parratt’s formula.^49^ The X-ray penetration is, therefore, the limiting factor for the probe depth as the escaping Fe 2p_3/2_ photoelectrons at that energy have longer IMFP equal to 4.87 nm.^50^ Hence, in the experiment, we measure a thin surface region of a few tens of atomic layers deep.

### Two Types of Iron Carbides

**Figure 3:**
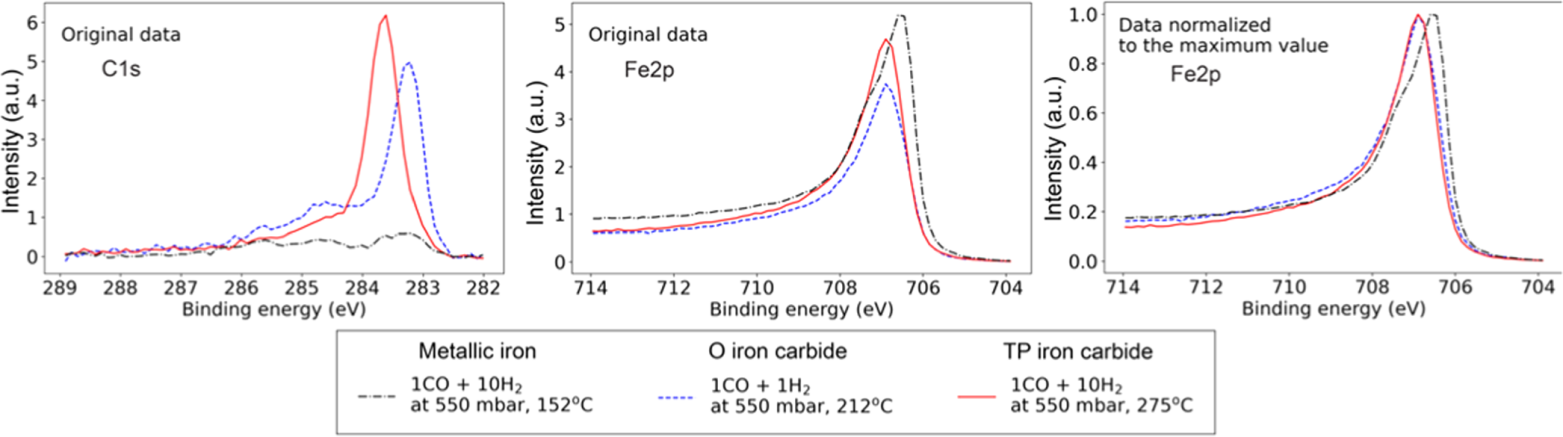
Photoelectron spectra of C 1s and Fe 2p_3/2_ regions recorded using 4.6 keV photons in 1CO:1H_2_ at 212 °C (blue dashed line) and in 1CO:10H_2_ at 152 and 275 °C (black dash-dotted and red solid lines). The left and middle panels show the C 1s and Fe 2p_3/2_ original data treated in the same way as all other data reported in the current contribution. The right panel shows Fe 2p_3/2_ data with every spectrum normalized to its maximum value for ease of comparison. “TP” and “O” indicate iron carbide structures with trigonal prismatic and octahedral sites occupied by carbon atoms, respectively.

Based on the transformation of the Fe 2p spectrum from the metallic multiplet to a single carbide peak together with the growth of the C 1s 283.3 and 283.6 eV signals, these latter two peaks were assigned to two types of iron carbides. Figure 3 shows that we were able to see these two types of iron carbides independently of each other under different conditions.

### Theoretical Calculations

To better understand the nature of the species involved in the observed process, we performed density-functional theory (DFT) calculations. Due to the specifics of different exchange–correlation functionals, the BE difference (ΔBE), also known as chemical shift, for the same element in different chemical environments is usually calculated instead of the absolute BE. Table 1 shows the computed values of ΔBE for iron carbide structures provided in the Materials Project database.^37^ To interpret the results, we separated all iron carbide structures into two groups: one in which the carbon is bonded to Fe atoms in an octahedral or slightly distorted octahedral (O) geometry and the other where carbon is bonded in a trigonal prismatic geometry (ΤP). O-carbides are associated with η-, ε-, and ζ-phases and ΤP-carbides—with Θ- and χ-phases.^8,51,52^ The most commonly detected carbides in the low-temperature (below ∼250 °C) Fischer–Tropsch synthesis are ε-Fe_2_C and ε′-Fe_2.2_C species from the O-carbide group, while at higher temperatures, the most often observed compounds are Θ-Fe_3_C, χ-Fe_5_C_2_, and Fe_7_C_3_, which exhibit TP geometry.^7−9,53^

**Table 1:**
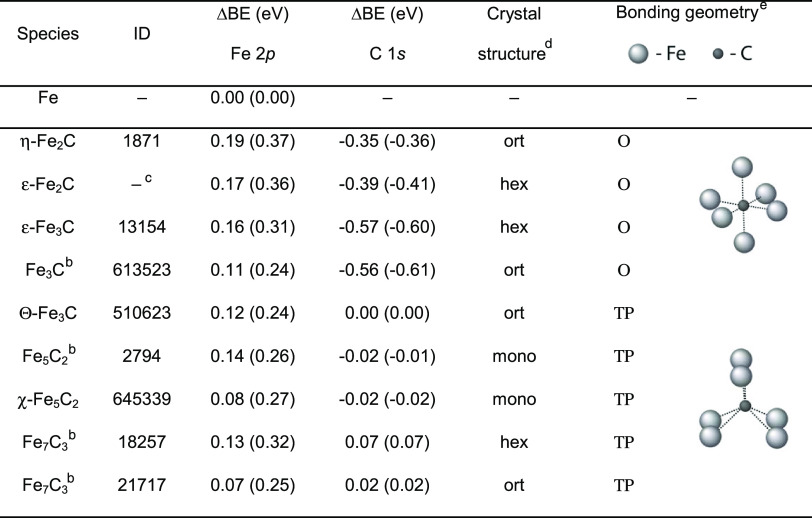
ΔBE Values for Fe 2p and C 1s Core Levels for Different Iron Carbides Identified by Their ID in the Materials Project Database^37^^a^^–^e

a Results are given for the core hole and, in parentheses, for the Z + 1 approximation. For the Fe 2p level, the BE shifts are relative to metallic iron, while for C 1s, they are relative to cementite Θ-Fe_3_C (ID 510623).

^b^ No phase classification available.

c Structural parameters were taken from a different source.^38^

d ort, orthorhombic; hex, hexagonal; mono, monoclinic.

e O, octahedral geometry; ΤP, trigonal prismatic geometry.

Table 1 shows that all calculated carbide Fe 2p ΔBE shifts from the metallic reference are within 0.10–0.20 eV for the core-hole approximation, while the corresponding Z + 1 values fall between 0.24 and 0.37 eV. This is fully consistent with the single carbide-related XPS Fe 2p peak observed experimentally (see Figure 3) since the spread of the values is only about 0.1 eV. The absolute values of the shift referenced to the metallic iron calculated using the Z + 1 approximation are close to the experimental result of 0.4 eV, while the corresponding values in the core-hole calculations are somewhat smaller. Atomic spin–orbit interactions largely cancel out when computing ΔBE. This minor difference can thus be related to the fact that the explicit core-hole calculation only considers the lowest resulting multiple, while the Z + 1 approximation, which does not include an explicit core hole, yields an average of the 2p final states, in this case, closer to the experiment.

In the case of C 1s BE shifts, the core hole and the Z + 1 approximations yield similar results, suggesting the presence of two distinct peaks for carbon atoms bound to iron in O (at lower BE) and ΤP geometries (at higher BE) separated by approximately 0.3–0.6 eV. This result is consistent with the experimental observations of 0.3 eV separation between carbide-related XPS C 1s peaks.

Based on the results of our experimental observations and DFT calculations, it is reasonable to speculate that at lower temperatures the initial carburization of the surface proceeds via the formation of one or several O-carbide phases with octahedrally coordinated carbon atoms contributing to the XPS C 1s peak at 283.3 eV. The peak at 283.6 eV may correspond to one or several ΤP phases that have close-lying C 1s BE and are more stable at higher temperatures. Both these phases would contribute to the single XPS Fe 2p peak shifted to higher BE relative to the metallic Fe 2p reference.

### Other Surface Species

The broad higher BE contribution that spans the region between approximately 283.8 and 285.8 eV (Figure 2; C 1s), where a multitude of species characteristic of the FTS process is expected, increases at higher temperatures. It is generally assigned to a combination of CH*_x_* fragments, longer-chain HCs, and surface-passivating carbon-containing phases, like sp^2^- and sp^3^-hybridized (graphitic and amorphous) carbon or coke or both.^5,6,46,54−57^ Graphene as a particular type of graphitic carbon phase may also be formed on iron surfaces.^58,59^ It is important to underline that due to the significant width of the signal (∼2 eV) it cannot be explained by a single compound since in such a case the expected contribution would be at least twice as narrow as the peak we observe.^59,60^

A shoulder at 531.3 eV in the O 1s region at low temperatures has been assigned to OH adsorbed on the surface, which is a regular byproduct in FTS.^6,61,62^ No significant signal characteristic for molecularly adsorbed CO around 285.9 and 532 eV may mean that the cleavage of CO molecules is rather fast, suggesting little or no contribution from the CO insertion mechanism to the reaction path under the examined conditions.^10^ This conclusion is also supported by the fact that the fraction of iron carbide and other carbide-containing species constantly grows, meaning that the dissociation of CO molecules is faster than the product formation and, thus, creates an excess of free carbon atoms.

### Time Dependence

The development of carbon-containing phases in general, and iron carbides, in particular, depends not only on temperature but also on the exposure time since the surface migration and permeation of carbon atoms into the bulk are kinetic processes that are especially facile on the {110} facets.^63^

**Figure 4:**
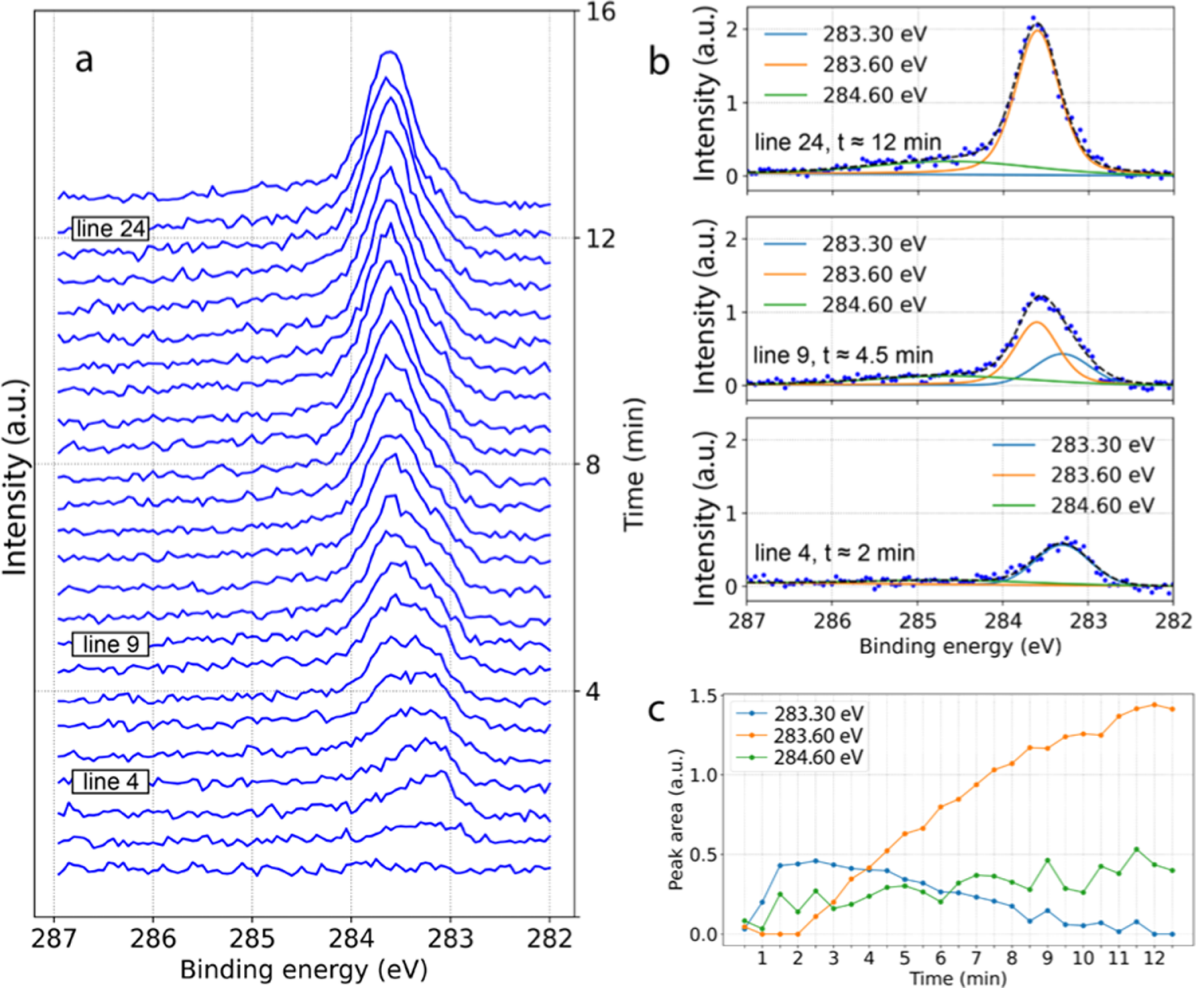
(a) Set of XP spectra of the C 1s region recorded using 4.6 keV photons for an Fe(110) single-crystal surface heated to 233 °C and exposed to 1CO:4H_2_ gas mixture at a 2 L*_n_*/min total flow and a 200 mbar pressure. Each spectrum took about 30 s, and the whole set of 25 spectra was accomplished in approximately 12.5 minutes; (b, c) examples of the fitting for lines 4, 9, and 24 corresponding to 2, 4.5, and 12 min experimental time, respectively, and peak area trends for the whole sequence in panel (a).

Figure 4a shows a continuous measurement of the C 1s region for an Fe(110) surface exposed to a 1CO:4H_2_ gas mixture at 200 mbar and a constant temperature of 233 °C. The recording of the C 1s region was started simultaneously with the gas exposure. The resulting sequence begins with a featureless spectrum indicating the absence of any carbonaceous deposits or carbon-containing reaction intermediates within the sensitivity of the instrument. However, immediately after the beginning of gas exposure, the component at 283.3 eV BE (O-carbides) starts to grow, and already after the fourth spectrum (approximately 120 s), it reaches its maximum and the component at 283.6 eV BE (ΤP-carbides) takes over and continues to grow slowly, replacing the initial signal. It should be noted that the exact time values presented in this section are valid for the particular experiment and do not translate into the quantitative determination of the carbide growth rate. The experimental complexity results in a number of factors causing the temporal variations between different measurement sets and, therefore, limiting the quantification capabilities. This, however, does not devaluate the observed general trends that hold true across all measured data sets.

In Figure 4b,c, the growth dynamics of observed species with time is demonstrated. The higher BE part of the spectra featuring a number of carbon-containing species as discussed above is represented here by one peak at 284.6 eV with 1.8 eV width. This higher BE contribution also grows in intensity but much slower.

The here reported observation of two types of iron carbides growing with time under FTS conditions at elevated pressure is in stark contrast to a previous ex situ study of thick iron films with the preferential (110) surface orientation carburized by cycles of exposure to atomic carbon or ethylene and annealing to the same temperature.^57^ In that work, the authors see the formation of a pure octahedral carbide phase, which reaches carbon saturation at about 15 atom % and does not detect tetrahedrally surrounded carbon atoms. Though the samples are prepared differently, the discrepancy is likely caused by the high pressure or the impact of the ongoing reaction or both as studied here and illustrates the importance of in situ studies of FTS systems.

### Pressure Dependence

At a 700 mbar pressure (Figure 2, bottom), the reduction of oxide and the development of carbon phases on the surface are shifted toward higher temperature or require a much longer time or both in comparison with the same processes at 85 mbar. The transition from oxidized to carburized state of the surface is observed at 212 and 233 °C for 85 and 700 mbar, respectively. What is also notable is that the formation of long-chain HCs and graphite/coke phase, as well as the transition between the two types of iron carbides, is significantly suppressed at higher pressure. This is evidenced by the surface being in a similar state at 275 and 318 °C for 85 and 700 mbar pressures, respectively. Another important observation is that the O-carbide is entirely gone and replaced by ΤP-carbide already at 318 °C at 85 mbar, while at 700 mbar, the contributions of both carbide types are similar in magnitude at the same temperature.

Such temperature shifts upon an increase of pressure are observed for all gas mixtures. Since the carburization of iron and the growth of other carbon-containing phases are driven by the free carbon atoms remaining unconsumed by the reaction after splitting of CO molecules, the observed trend likely points at a more efficient reaction process at higher pressures allowing for higher consumption rate of carbon atoms. It is not unexpected since industrial FTS reactors usually operate at pressures of around 10 bar and higher. It is at the same time satisfying to directly observe the effect of pressure on the reaction efficiency with an in situ surface-sensitive experimental technique.

An alternative explanation of the retarded growth of carbon-containing species on the surface at higher pressures could be a pressure-dependent change of the surface coverage ratio of H_2_ to CO. That is, the ratio of the surface chemical potential of the reactants is changing with pressure at otherwise identical conditions. This possibility is supported by the fact that also the removal of oxide is retarded with increasing pressure, indicating the decrease of surface reduction rate driven by CO.

Another important observation is that the O-carbide phase seems to be more stable at higher pressure and may, therefore, be present in larger amounts in real catalysts even at higher temperatures, where it is supposed to be less stable and thus playing an important role in the catalytic process. Recently, ε-Fe_2_C (O-type carbide) was stabilized in the high-temperature FTS process by means of structural confinement and was shown to be superior in activity than other types of iron carbides.^64^

### CO-to-H_2_ Ratio Dependence

**Figure 5:**
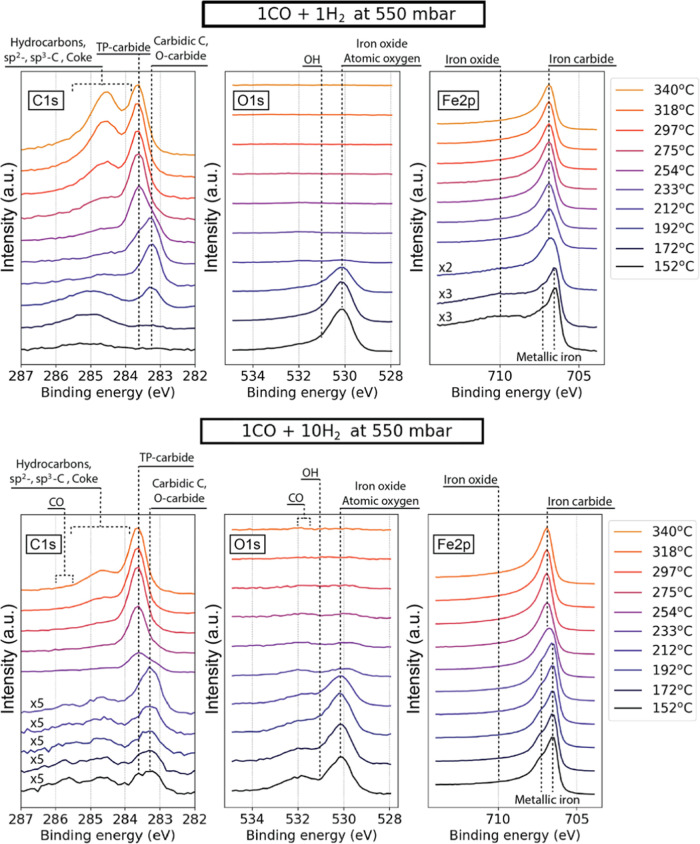
Photoelectron spectra of C 1s, O 1s, and Fe 2p_3/2_ regions recorded using 4.6 keV photons in 1CO:1H_2_ (top row) and 1CO:10H_2_ (bottom row) reaction gas mixtures at 2 and 2.5 L*_n_*/min total flows correspondingly and 550 mbar pressure. Note that selected lower-temperature spectra of C 1s and Fe 2p_3/2_ regions are magnified by factors of 2, 3, and 5 for better visibility of the iron oxide contribution and surface carbon-containing adsorbates. Approximate locations of determined species are annotated in the figure. “TP-” and “O-carbide” stand for carbide structures with trigonal prismatic and octahedral sites occupied by carbon atoms, respectively.

Figure 5 shows a comparison of the surface evolution in 1:1 and 1:10 gas mixtures of CO and H_2_ at 550 mbar pressure, respectively. In the 1:1 reaction mixture, the development of the surface resembles the behavior in the 1CO:4H_2_ mixture at 85 mbar, with the difference that the transition from oxidized to carburized state of the surface occurs at an even lower temperature and occurs already at 192 °C. Other differences that are worth noting are a much faster transition between the iron carbide phases at 283.3 and 283.6 eV BE. The iron carbide transition occurs between 233 and 254 °C for a 1:1 gas mixture and between 233 °C and almost 297 °C for a 1:10 gas mixture. It should be noted that a somewhat elevated amount of amorphous and graphitic carbon is present between approximately 284.5 and 285.5 eV BE at low temperatures in a 1:1 reaction mixture, likely due to dissociation of the abundant CO molecules.

In the hydrogen-rich reaction mixture, the Fe 2p_3/2_ region is metallic from the beginning with no detectable oxide component. The O 1s region still features a signal at 530.1, which in this case is weaker and entirely comprised of adsorbed surface oxygen.^61^ In contrast to all other gas mixtures and pressure regimes, this data set features a detectable contribution from adsorbed CO at around 532 and 285.8 eV, which is still small and disappears above ∼220 °C. The signal from long-chain HCs and graphite/coke phase is significantly smaller than for all other conditions at all temperatures. These observations are in line with the literature reporting a higher conversion rate of the reaction and slower carburization process.^65−67^

### Mass Spectrometry Measurements

**Figure 6:**
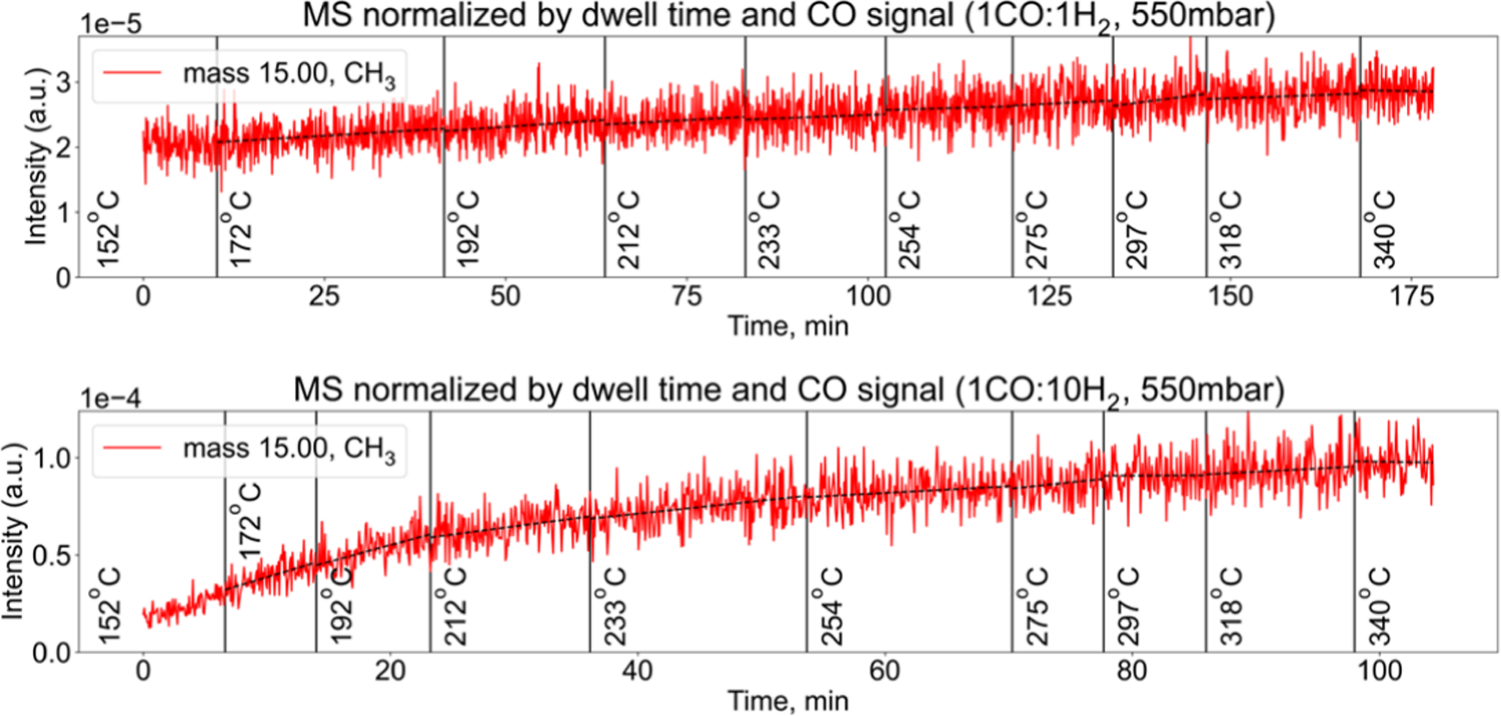
Mass spectrometry signal corresponding to the sets of measurements in 1CO:1H_2_ (top) and 1CO:10H_2_ (bottom) gas mixtures at a 550 mbar pressure. The signal is normalized by the dwell time and respective CO signal to eliminate the effect of potential total pressure fluctuations and drift in the mass spectrometer. The broken lines in both panels show the linear fit of every temperature interval.

No reaction products were observed by XPS in the gas phase in the C 1s and O 1s regions. At the same time, we observe a weak but clear increase of the CH_3_ (methyl) radical signal in the mass spectrometry, which indicates a hydrogenation reaction on the surface. Figure 6 shows the recorded signal corresponding to the set of measurements in the 1CO:1H_2_ and 1CO:10H_2_ gas mixtures at 550 mbar. In the figure, we note a steeper increase in signal for the more hydrogen-rich 1:10 gas ratio than for the 1:1 mixture. A higher partial pressure of H_2_ is known to impact the HC termination reaction, resulting in higher activity overall, and specifically for shorter HCs such as methane. Furthermore, the higher content of long-chain HCs and graphite/coke observed by XPS (see Figure 5) likely contributes to the passivation of the surface for lower H_2_/CO gas ratios, resulting in lower activity. It is essential to remember that typical activation times for FTS catalysts greatly exceed the acquisition times for the XP spectra shown in this work. As a consequence, the MS signal increases even between the temperature steps. It should also be noted that the mere increase of the signal does not tell us whether the catalyst’s activity is high or low on the absolute scale. However, it sufficiently confirms that the CO is being converted to hydrocarbons and that this process is more active at higher hydrogen partial pressures.

### SXRD Experiment

After the sample cleaning, the reactor volume was isolated from the UHV part and filled with pure H_2_ while the sample was cooled to 150 °C. Subsequently, a mixture of H_2_ and CO was guided into the reactor in a controlled way, allowing for changes in the partial pressure of the reactants. The total gas pressure around the sample was set to 150 mbar and controlled by a Bronkhorst back-pressure controller. The temperature of the sample surface was increased stepwise from 150 to 350 °C, while the diffraction patterns and the mass spectrometry signal were recorded continuously. The latter resembles the data shown in Figure 6 and is thus not shown here.

**Figure 7:**
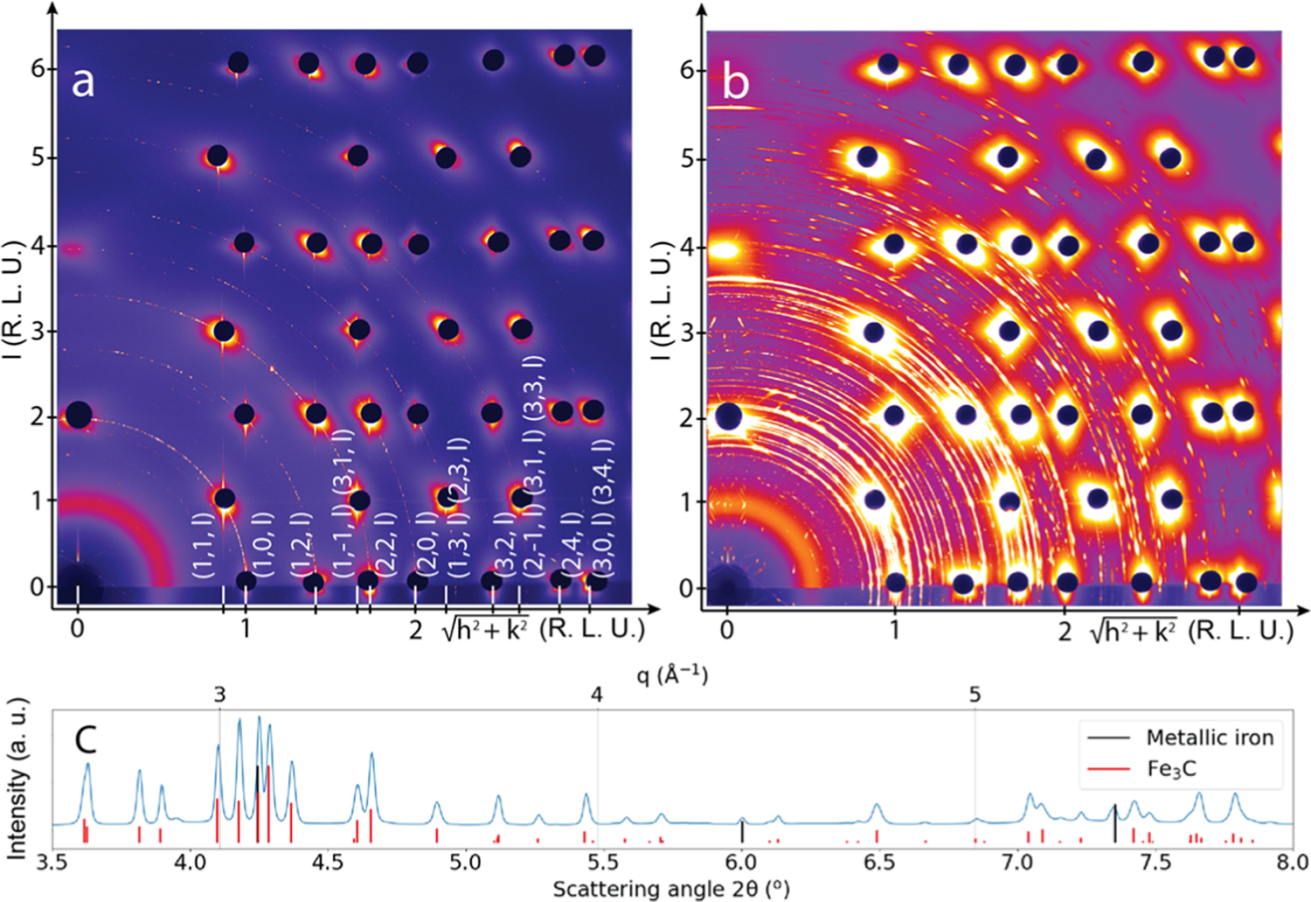
2D diffraction patterns recorded using 83 keV photons for (a) the metallic Fe(110) surface in pure H_2_ at 150 °C after the cleaning procedure and (b) the Θ-Fe_3_C-covered surface at 350 °C after a set of measurements in a 1CO:4H_2_ reaction gas mixture at a 150 mbar total pressure; (c) circularly integrated representation of the 2D pattern in panel b, with the indicated reference values for metallic iron (black lines) and Θ-Fe_3_C (red lines). Axes in panels (a) and (b) are given in reciprocal lattice units (RLUs) (see Section S2 in the Supporting information for more details).

A diffraction pattern characteristic for the surface prior to each set of measurements is shown in Figure 7a. It represents a clean metallic surface based on the presence of crystal truncation rods (CTRs) corresponding to a (110)-oriented body-centered cubic surface (see Section S2 in the Supporting Information for details of SXRD data processing and CTR indexing). Weak polycrystalline rings also visible in the pattern are likely to arise from the edges of the crystal where the sputtering procedure is less efficient. Figure 7b shows the state of the surface at 350 °C after a full set of measurements in a 1CO:4H_2_ reaction mixture. Seeing intense diffraction rings, it becomes immediately clear that the surface became rough and polycrystalline. By circular integration of the two-dimensional (2D) pattern, it is possible to present the data in the conventional form of diffracted intensity versus scattering angle. Figure 7c shows such a representation with the addition of vertical lines showing the reference values for metallic iron (in black) and Θ-Fe_3_C (cementite) compound (in red).^68^ Comparing the diffraction pattern to the references of other iron carbide and oxide compounds, graphite shows that no such species are present on the surface.

Performing the diffraction experiment, we observed the direct transition from the oxidized state of the surface upon entry of the reaction gas mixture to the metallic state and further to the formation of Θ-Fe_3_C compound without any other intermediate iron carbide species. These findings hold for other reaction mixtures studied in the diffraction experiment, namely, 1CO:1H_2_ and 1CO:10H_2_ (see Section S7 in the Supporting Information for data examples).

Although diffraction experiment probing depth is larger than the probing depth in the XPS experiment, we are confident that the results of two experiments can be compared. The estimated probing depth for XPS is a few tens of atomic layers, as discussed earlier. In multiple cases at various temperatures and gas compositions in XPS (e.g., 275 °C in a 1CO:4H_2_ reaction gas mixture at all investigated pressures or at 212 °C and 233 °C in 1CO:1H_2_ and 1CO:2H_2_ gas mixtures at all investigated pressures), we see the coexistence of both O and ΤP types of carbides with a similar contribution to the C 1s photoelectron spectra, while the corresponding Fe 2p spectra show the complete carburization of iron. At the same time, the feature at higher binding energy in the C 1s region corresponding to other carbon-containing deposits is smaller in comparison with the carbide signal. This means that a tens-of-atomic-layer-thick surface region consists, to a large extent, of two types of iron carbides in similar quantity. It has been shown that surface structures with a fraction of monolayer coverage can be distinguished in SXRD;^19,20^ thus, both carbides should be possible to see in the diffraction experiment at the corresponding conditions.

If one now compares the SXRD and XPS experimental results, it can be concluded that the ΤP-carbide phase found in the photoelectron spectra is likely to be related to the Θ-Fe_3_C (cementite) compound, while the O-carbide phase was not observed by diffraction. While the latter variation in detection between the two separate experiments may be caused by the difference in some experimental factors, it is also possible that the O-carbide phase observed in XPS can be lacking a strict structural order, which could be explained by the permeation of carbon atoms into the iron lattice resulting in random occupation of the naturally available octahedral sites within the body-centered cubic structure. The trigonally prismatic coordination of the carbon atoms, on the other hand, requires a major reconstruction of the original iron lattice, thus prompting the formation of a new carbide phase with a new distinct structural order. Such an explanation would imply the possible presence of a disordered iron–carbon octahedral phase that may play an important role in the reaction process while staying invisible to diffraction-based experimental techniques. Further theoretical studies of the FT process involving a disordered carbide phase could shed more light on this issue and may be essential for a full understanding of the catalytic process.

## Conclusions

We report direct spectroscopic and diffraction observations of the effect of pressure (up to 700 mbar) and gas feed composition on the FTS reaction efficiency and the surface state of a model Fe(110) single-crystal catalyst with an in situ surface-sensitive spectroscopic experimental technique. In XPS, we observe two distinct types of iron carbides determined as O-carbides (octahedral) and ΤP-carbides (trigonal prismatic) growing on the surface as a function of both time and temperature. Comparing different pressures and gas compositions, we outline several qualitative trends. First, comparing the same gas mixture at low and high pressures, the growth of all carbon phases is suppressed and delayed to higher temperatures at higher pressures, which likely means that it takes longer time for free carbon atoms to accumulate on the surface at higher pressures, pointing either to a faster conversion of reactants to reaction products or to the pressure-dependent change of the ratio of surface chemical potential of the reactants. Second, the O-carbide signal at 283.3 eV BE always appears first and starts to decrease when the ΤP-carbide signal increases with both temperature and time. Third, the multicomponent signal at higher BE-containing reaction products and surface-passivating carbon-containing deposits decreases relative to the total spectral area with both the increase of the pressure and the increase of the hydrogen content at all recorded temperatures. The overall absence of any significant amount of the molecularly adsorbed CO on the surface (except the case of 1CO:10H_2_ at temperatures lower than ∼220 °C) may mean that the CO insertion step is little or not at all involved in the FTS processes under almost all examined conditions.

The diffraction studies show the formation of Θ-Fe_3_C (cementite, ΤP-phase) in the active state of the catalyst while no sign of the O-phase formation is seen, which may be due to a disordered nature of the O-type species observed in XPS. If that is the case, the nonordered octahedrally coordinated carbon atoms may be an important source of carbon for the catalytic reaction. Additionally, no other ordered structures, like graphite or other iron carbides, were detected in the diffraction experiment.

## Supporting Information Available

PDF file contains details of (i) experimental methods, (ii) sample preparation and assessment, (iii) the beam damage assessment, (iv) sample heating and surface temperature measurements, and (v) XPS data corrections; it also contains more data sets for the readers’ reference (PDF)
