# Supplementary material for: In Situ Surface-Sensitive Investigation of Multiple Carbon Phases on Fe(110) in the Fischer–Tropsch Synthesis
Source: ACS Catal. 2022 Jun 13;12(13):7609–21. doi: 10.1021/acscatal.2c00905 (PMC9254136; doi:10.1021/acscatal.2c00905)
Supplement: Supplementary file 1 — cs2c00905_si_001.pdf [file cs2c00905_si_001.pdf]

In situ surface-sensitive investigation of multiple carbon phases on Fe(110) in Fischer-Tropsch synthesis

Mikhail Shipilin[1]*, David Degerman[1], Patrick Lömker[2], Christopher M. Goodwin[1], Gabriel L.S. Rodrigues[1], Michael Wagstaffe[3], Jörgen Gladh[1,4], Hsin-Yi Wang[1], Andreas Stierle[3,5], Christoph Schlueter[2], Lars G.M. Pettersson[1], Anders Nilsson[1]*, Peter Amann[1]

[1] Department of Physics, Stockholm University, 10691 Stockholm, Sweden

[2] Photon Science, Deutsches Elektronen-Synchrotron DESY, 22607 Hamburg, Germany

[3] DESY NanoLab, Deutsches Elektronen-Synchrotron DESY, 22607, Hamburg, Germany

[4] PULSE Institute, SLAC National Accelerator Laboratory, Menlo Park, 94305 California, USA

[5] Physics Department, University of Hamburg, 20148 Hamburg, Germany

* Corresponding authors: andersn@fysik.su.se, mikhail.shipilin@fysik.su.se

Abstract: Carbide formation on iron-based catalysts is an integral and, arguably, the most important part of the Fischer-Tropsch (FT) synthesis process converting CO and H_2 into synthetic fuels and numerous valuable chemicals. Here we report an in situ, surface-sensitive study of the effect of pressure, temperature, time and gas-feed composition on the growth dynamics of two distinct iron carbide phases with octahedral and trigonal prismatic coordination of carbon sites on a Fe(110) single-crystal acting as a model catalyst. Using a combination of state-of-the-art X-ray photoelectron spectroscopy at unprecedentedly high pressure, high-energy surface diffraction, mass-spectrometry, and theoretical calculations, we reveal the details of iron surface carburization, and products formation under semi-realistic conditions. We also provide a detailed insight into the state of the catalyst's surface in relation to the reaction.

Table of Contents

S1. Materials and methods....2

S2. Sample preparation....3

S3. X-ray beam induced effects....6

S4. Sample heating and surface temperature measurements....8

S5. Fermi level correction....9

S6. XPS experimental data examples....10

S7. Diffraction experimental data examples....15

S8. Example of VASP INCAR file for calculating 1s and 2p binding energy....17

S1. Materials and methods

XPS experimental setup. In this work we employ the POLARIS instrument at the P22 beamline of the PETRA III synchrotron at DESY.[1–3] It is based on a Scienta-Omicron R4000-HiPP-2 electron analyser and is designed to be capable of acquiring photoelectron spectra at pressures from several hundred mbar to 1 bar and beyond.
The spectra were recorded using photons at 4.6 keV energy that were impinging on the surface at an incident angle of 0.4 degrees with respect to the surface plane. This is below the angle of total external reflection for iron at that energy. High energy increases the photoelectron mean free path while low incident angle enhances the surface sensitivity.[1] The pass-energy of the electron analyser was 100 eV. The incoming beam was focused down to ca. $10\times15\ \mu m^2$ with the photon flux of about 10^{13} photons per second distributed over the entire footprint, which at this angle was about $1400\times15\ \mu m$ yielding about 4.8×10^{14} photons/s per 1 mm^2.
The sample sits in a custom-made holder manufactured from 1.4762 - AISI 446 steel and heated with a BN heater, which is in direct contact with the backside of the crystal. Due to the specific design of this experimental setup, no contamination from the heater or chamber can reach the sample surface when gas flow is applied.

SXRD experimental setup. The diffraction experiments were performed using synchrotron radiation with an energy of 83 keV at the P21 beamline of PETRA III, Deutsches Elektronen-Synchrotron (DESY) in Hamburg, Germany. The details of the High-Energy Surface X-Ray Diffraction methodology, data recording and treatment can be found elsewhere.[4,5]
The sample was placed in a specially designed chamber[6] mounted on a Huber® diffractometer stage, which allows for a precise surface alignment to the incident beam. In order to maximize the surface to bulk signal ratio, the angle of incidence was set to 0.035º, which is slightly above the critical angle of total external reflection for iron when irradiated by 83 keV X-rays. The chamber combines an ultra-high vacuum (UHV) part for sample preparation and a 50 ml gas flow reactor in which the sample can be exposed to a gas mixture.
During the experiment the sample was continuously exposed to a photon flux on the order of $1.3\cdot10^{10}$ photons/sec with an incident beam size of 4x10 μm^2. The beam induced effects were tested and found to not influence the experiment.
To record the diffraction patterns a 430x430 mm^2 Varex Imaging flat panel X-ray detector was used. It has a resolution of 2880x2880 pixels with the physical size of 150x150 μm^2. In order to avoid the saturation by highly intense diffraction maxima, the corresponding parts of the detector were covered with Tungsten pieces blocking the electromagnetic radiation. This protection is seen in experimental patterns as black circles.

Mass-spectrometry. To measure the reaction products in the XPS experiment, a Hiden mass spectrometer (HAL/3F RC 301 PIC system) attached to the differential pumping of the spectrometer was used. It allows for sensing the gas flowing directly from the reaction volume to the pump through the aperture of the analyser's nozzle. Thus, XPS and MS data are from an as close as possible probing volume. In the SXRD experiments, the partial pressures of the reactants and the reaction products were followed with a RGA200 Stanford Research Systems residual gas analyzer accessing the reaction volume via a leak.

Recoil effect in XPS data treatment. Using the approximation described by Takata et al.[7] it is possible to estimate the magnitude of the shift and the broadening caused by the recoil effect for, respectively, C 1*s*, O 1*s* and Fe 2*p* spectral lines measured at 4.6 keV photon energy, i.e. the values relevant to the present work.
The energy shift can be calculated using a simple formula $\delta E = (m/M)\cdot E_{kin}$, where M is the mass of the atom, m is the electron mass, and $E_{kin}$is the photon energy. The broadening of the spectral lines, then, can be approximately estimated as $2\cdot\delta E\cdot k_B T$, where k_B is the Bolzman constant ($\approx 8.617\cdot10^{-5}[eV\cdot K^{-1}]$) and T is temperature in K.

S2. Sample preparation

In all experiments the surface of a 4N5 purity Fe(110) single-crystal purchased from Surface Preparation Lab (SPL) was prepared by multiple cycles of 30 min Ar^+ ion sputtering at 1.5 kV and annealing at up to 700°C for 5 minutes alternating with hydrogen treatment at 10-100 mbar pressure and 400°C temperature (occasionally the H_2 flow was exchanged with CO_2 flow for mild surface oxidation).

For XPS experiments, survey scans were acquired every time before and after a set of measurements in the experiment to ensure the absence of surface contamination. The main focus was set on checking for such common contaminants as silicon and sulfur. The carbon C 1*s* region was also required to be featureless, although, small amounts of adventitious carbon were tolerated if present since it is almost impossible to get rid of it outside of Ultra-High Vacuum (UHV) conditions. Besides, pure hydrogen flow ushering every measurement effectively removed adventitious carbon. A mild oxidation of the surface in the form of iron oxide was also not considered as a contamination since it is notoriously difficult to keep iron metallic outside UHV conditions and it inevitably oxidizes upon introduction to the chamber of a CO containing reaction gas mixture and reduces later in the carburization process.

In Figure S2-1, one can see two representative spectra taken at 100 mbar pressure of pure hydrogen at 152°C prior to the measurement and at 550 mbar pressure of the 1CO : $2H_2$ reaction gas mixture at 318°C after the corresponding experiment. The spectrum in the top panel taken in hydrogen, shows the absence of contaminations on the surface before the set of measurements. The spectrum at the bottom features the residual carbon signal in C 1*s* region and the signal from the gas phase CO in both C 1*s* and O 1*s* regions (see Figure S2-2 for more details). Note that a high background of photoelectrons inelastically scattered in the gas phase as well as additional electron energy loss features (H_2 excitation 12.8 eV, CO excitation 8.5 eV) are present in the spectra on the higher binding energy side of each peak due to the high gas pressure.

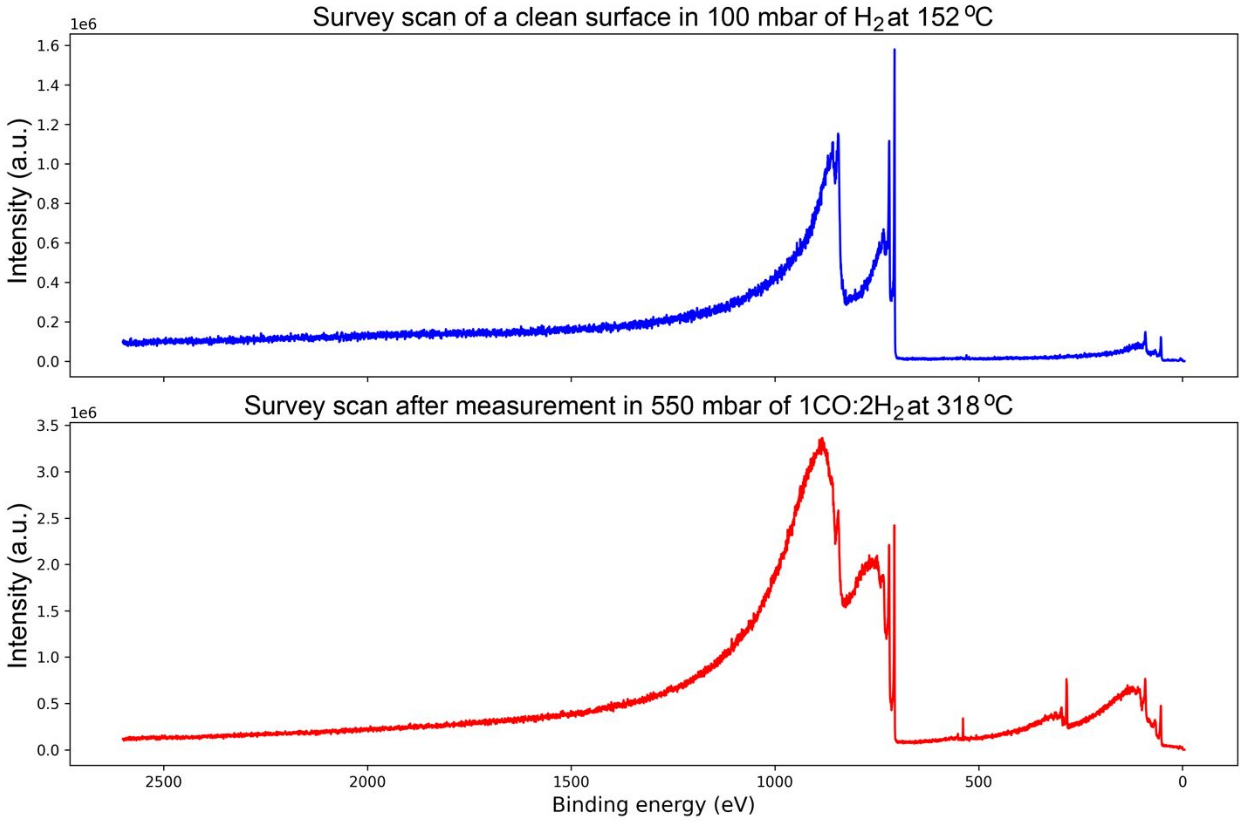

Figure S2-1. X-ray photoelectron survey spectra of the Fe(110) surface (top) before and (bottom) after the set of measurements at 550 mbar pressure recorded using 4.6 keV photons in 1CO : $2H_2$ gas mixture. The spectrum in the top panel was taken at 152°C in 100 mbar pure hydrogen, while the spectrum in the bottom panel was taken at 318°C in 550 mbar of the reaction mixture.

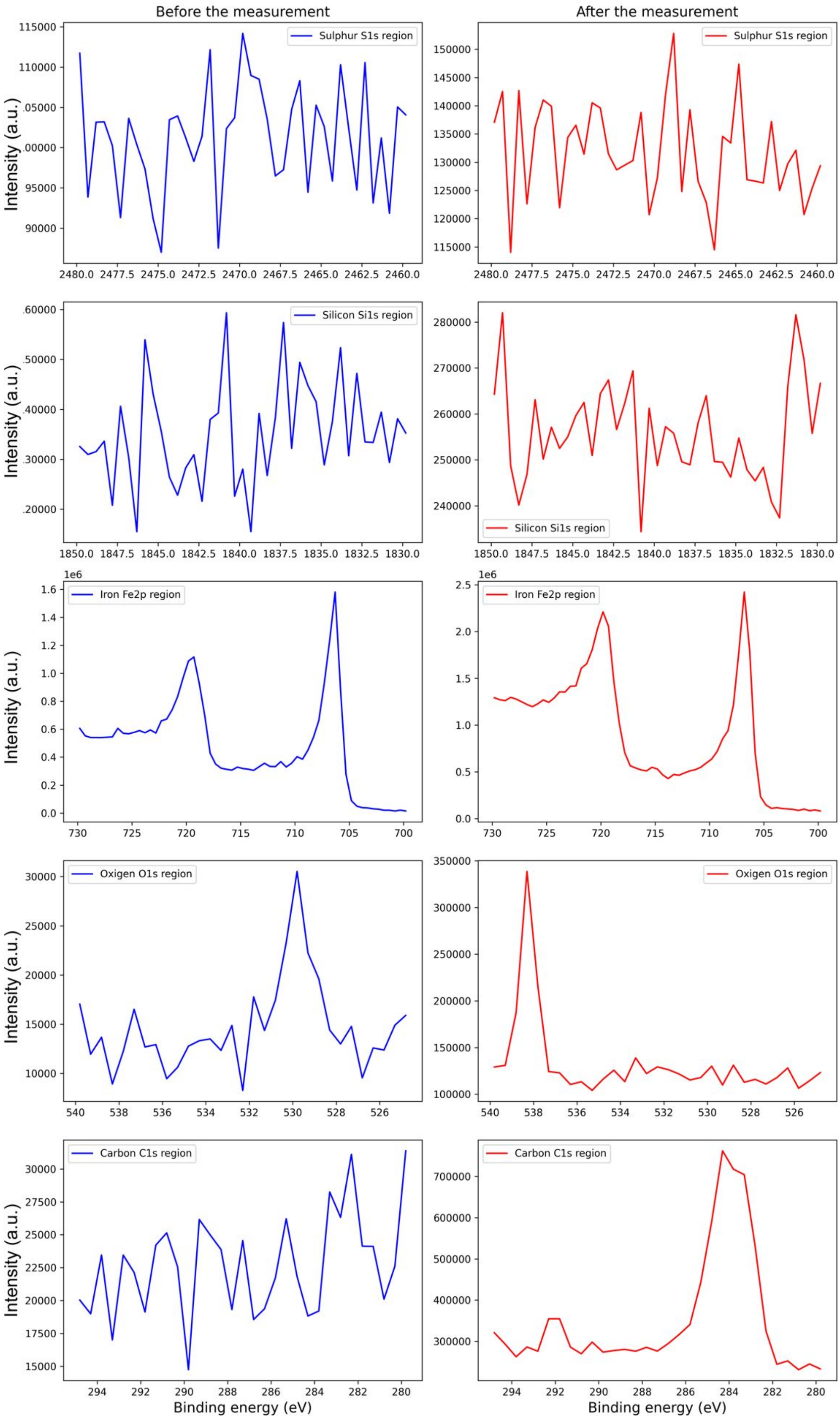

Figure S2-2. Enlarged fragments of X-ray photoelectron survey spectra of the Fe(110) surface (left column) before and (right column) after the set of measurements at 550 mbar pressure recorded using 4.6 keV photons in 1CO : $2H_2$ gas mixture. The spectra on the left were taken at 152°C in 100 mbar pure hydrogen, while the spectra on the right were taken at 318°C in 550 mbar of the reaction mixture.

For the diffraction experiment, the sample was prepared in the same manner as for the XPS studies. Before every set of measurements, a survey pattern was taken and was required to represent a flat metallic surface. One such pattern is shown in Figure S2-3, panel *d*. The black circular shapes represent the tungsten pieces covering the detector from oversaturation in the places where the diffraction maxima from the crystal should occur. The vertical streaks of enhanced intensity represent the crystal truncation rods originating from the Fe(110) surface. The insignificant traces of polycrystalline iron phase indicated by the weak rings in the diffraction patterns were assigned to the edges of the sample that could not be efficiently sputtered and were tolerated. The absence of a significant amount of disordered contaminants like Sulphur was implied based on the previous X-ray photoelectron spectroscopy studies of the same sample where a similar treatment did not cause any significant segregation of impurities on the surface.

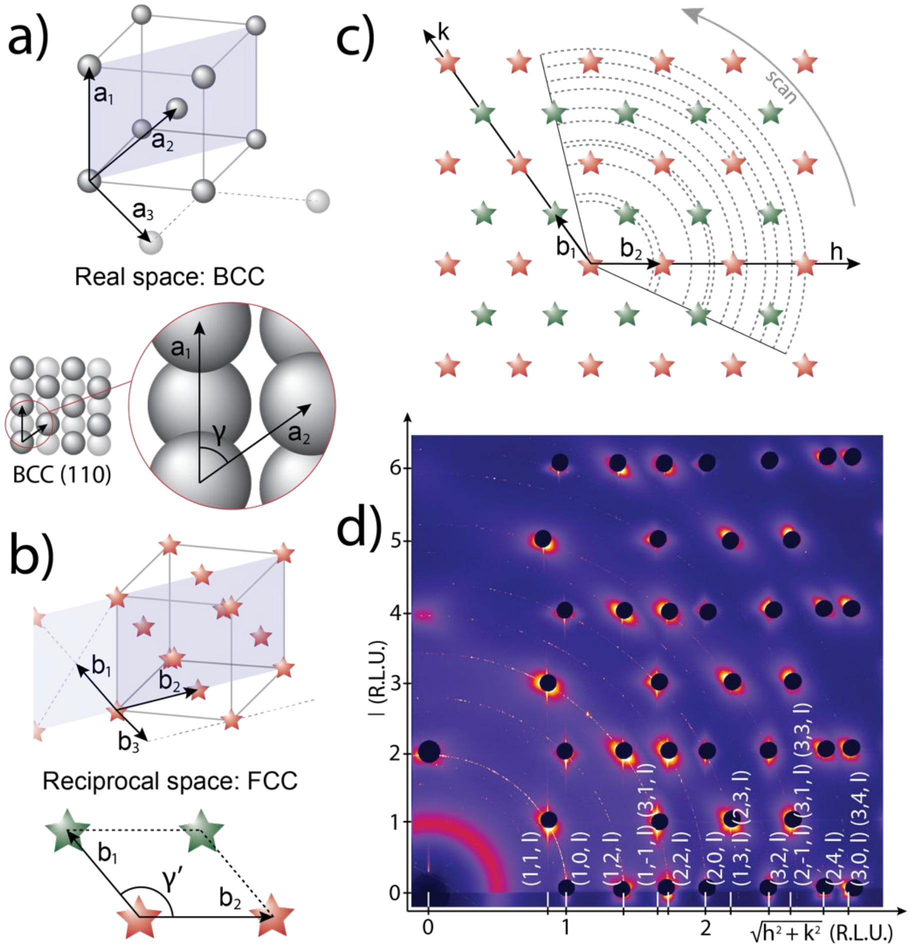

Figure S2-3. Schematic representation of (a) a body-centered cubic (BCC) unit cell, and a BCC(110) surface, (b) the corresponding face-centered cubic (FCC) reciprocal unit cell and its (110) plane, and (c) the hk-plane (in-plane) arrangement of the reciprocal lattice nodes for the (110) surface orientation. Note that basis vectors are given for (110) surface orientation and green and red stars represent the nodes in parallel planes at different l-values (out-of-plane values). (d) Experimental surface diffraction pattern recorded using 83 keV photons for a clean Fe(110) single crystal obtained by integration of a rotational scan indicated in panel (c) with in-plane indices given for respective surface truncation rods (STRs).

To describe the surface structure, a monoclinic basis set of vectors a_1, a_2 with the angle γ = 54.74° between them lying in the surface plane and a_3 perpendicular to them was used. In terms of the bulk iron lattice constant a_0 = 2.866 Å, the lengths of these vectors are $|a_1| = a_0 = 2.866$, $|a_2| = \frac{\sqrt{3}}{2} \cdot a_0 = 2.482$, and $|a_3| = \sqrt{2} \cdot a_0 = 4.053$ Å. The corresponding reciprocal unit cell has then its basis consisting of vectors b_1, b_2 with the angle γ' = 144.74° between them lying in the surface plane and b_3 perpendicular to them, with the lengths of 2.685, 3.100, and 1.550 $Å^{-1}$ respectively.

In the current experiment each recorded diffraction pattern corresponds to a vertical slice in the reciprocal space at a fixed sample azimuth and spans over the area from 0 to 10 $Å^{-1}$ in both in-plane and out-of-plane directions. In

the case of Fe(110) this part contains diffraction peaks with *h*-values up to 3, *k*-values up to 4, and *l*-values up to 6 Reciprocal Lattice Units (RLU). Diffraction data was collected by recording the diffraction patterns corresponding to different azimuthal angles during the sample rotation by 125º under the photon beam. The angular step was chosen to be 0.2º resulting in 625 diffraction images in one data set. To resolve the surface changes in time, separate measurements at a constant azimuthal rotational angle corresponding to the *hl*-plane in reciprocal space were performed as well recording the detector images every 5 seconds.

S3. X-ray beam induced effects

In order to ensure the absence of X-ray beam induced effects, we performed the following experiment. Two separate measurements were done under identical conditions (200 mbar pressure, 233°C temperature, 1CO:4H_2 gas mixture at 2.1 L_n/min total flow, incidence angle 0.4°) with the same initial clean state of the surface. In the first measurement, the surface was exposed to the X-ray beam and the surface evolution was recorded for 20 minutes. In the second measurement, the X-ray flux was set to zero by closing the beamline shutter and the surface stayed under the same conditions as in the previous measurement for 20 minutes. Then the beam was switched on and the state of the surface was recorded. Figure S3-1 shows the results of these measurements.

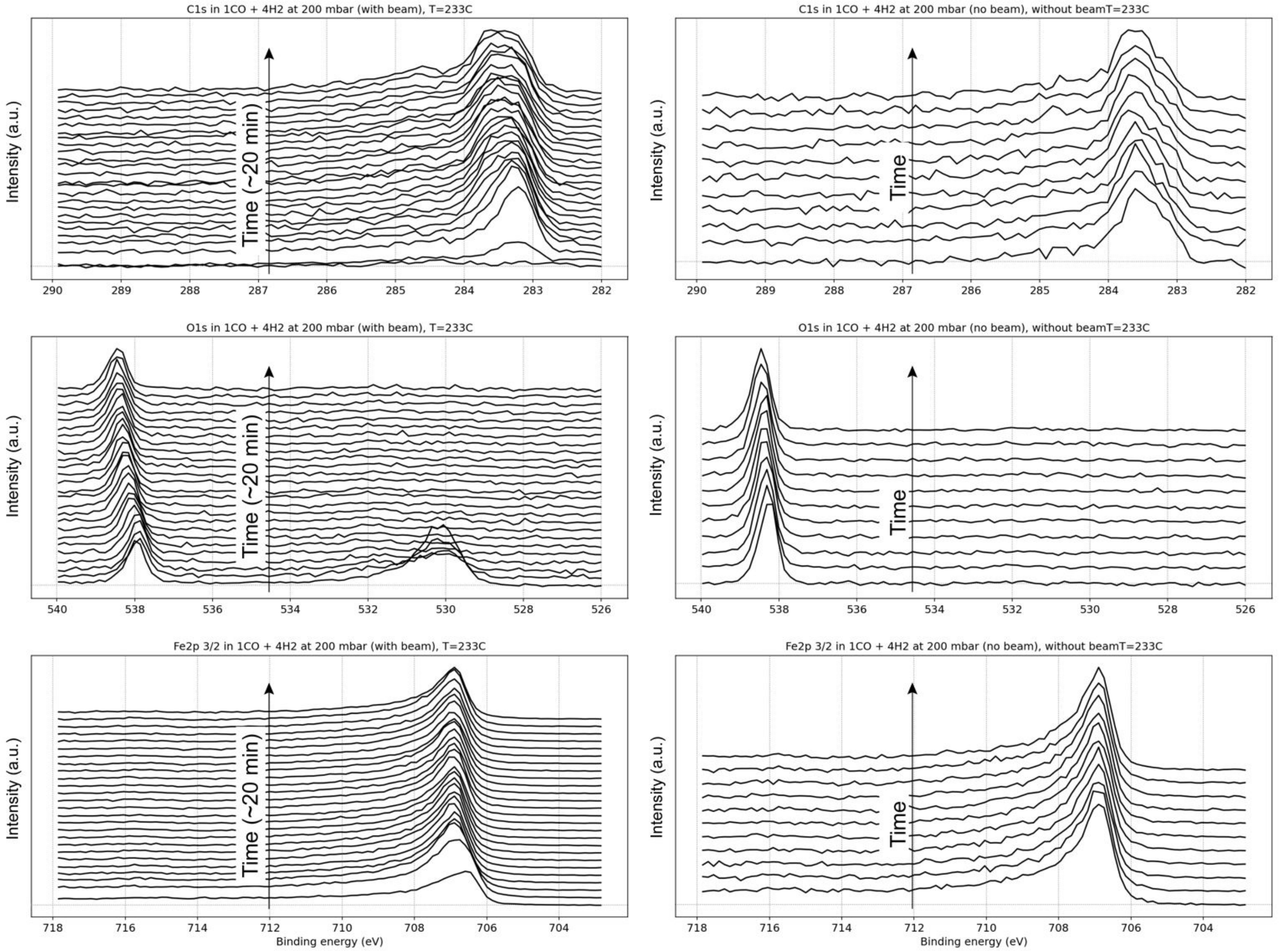

Figure S3-1. Time resolved XP spectra recorded using 4.6 keV photons for C 1*s*, O 1*s* and Fe 2$p_{3/2}$ levels of Fe(110) single crystal surface exposed to 1CO : 4H_2 gas mixture at 2.1 L_n/min total flow at 200 mbar pressure and 233°C temperature for 20 minutes. (Left column) with X-ray beam exposure from the beginning and (right column) after 20 minutes of no beam exposure.

As one can see from the left column of Figure S3-1, the first sweep of the measurement records iron oxide on the surface as always when the transition between UHV and high-pressure mode is done. At the same time no significant carbon signal is visible. Very quickly, the oxide is reduced and the C 1*s* signal reveals the appearance

and growth of carbon species between 283 and 284 eV binding energy. Later, also signal between 284 and 286 eV binding energy gets more pronounced. The right column shows the state of the surface after it had been exposed to the same conditions but without the photon beam for 20 minutes.
The logics of the experiment tells that if no beam-induced effects are involved, the last (topmost) sweep of each electron level in the left column should be similar to the first (bottommost) sweep of each corresponding level in the right column, as they both are meant to capture the surface after 20 minutes of exposure to the same reaction conditions. Naturally, these sweeps should be significantly different if beam-induced effects are involved. In Figure S3-2, we take a closer look at these two sweeps for C 1*s* electron level.

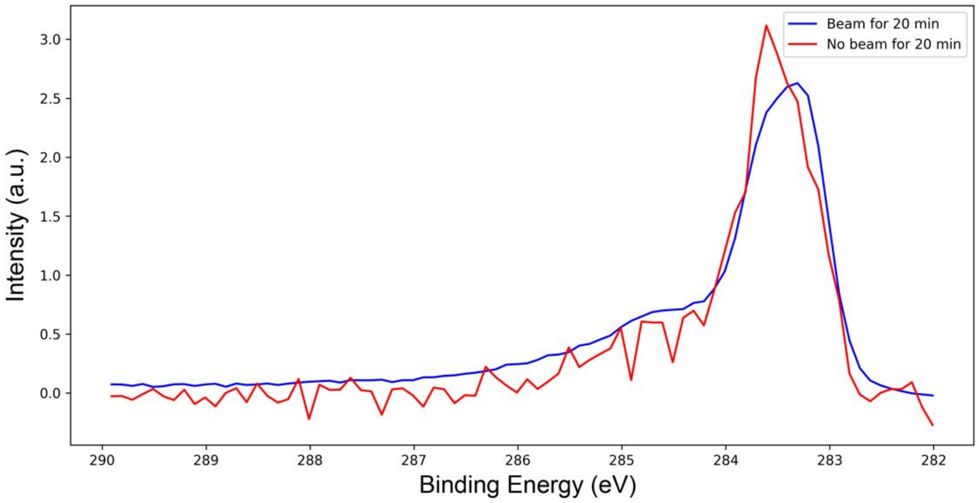

Figure S3-2. Comparison of C 1*s* XP spectra recorded using 4.6 keV photons for Fe(110) single crystal surface exposed to 1CO : $4H_2$ gas mixture at 2.1 L_n/min total flow at 200 mbar pressure and 233°C temperature for 20 minutes with and without photon beam.

From Figure S3-2 it becomes clear that even though the XP spectra don't look entirely equal, the same carbon species are present on the surface in a comparable amount. This means that the beam-induced effects are either non-existent or don't influence the principles of surface evolution under reaction conditions. A slight difference between the spectra could have been caused by a slight difference in the initial state of the surface, such as a presence of small amount of adventitious carbon, or a fluctuation in the gas flow – the experimental uncertainties that cannot be entirely avoided in such a complex experiment. It should be noted that the spectrum measured after 20 minutes of no beam exposure is noisier than the spectrum with the beam on. The reason for that is a small sample to beam misalignment that accumulates over time due to the thermal drift and is corrected by maximizing the signal that is being detected. Naturally, when the beam is disabled for 20 minutes, no such correction is being performed resulting in lower signal after the beginning of the exposure.
The corresponding O 1*s* and Fe 2$p_{3/2}$ spectra are identical and don't reveal any beam induced traces.
The same approach was taken in the diffraction experiment with the same result that did not show any significant difference between the diffraction patterns after keeping the sample for 20 minutes under the reaction conditions with the X-ray beam on and off.

S4. Sample heating and surface temperature measurements

In the XPS experiment, the sample rests in a custom-made holder manufactured from 1.4762 – AISI 446 steel and heated with a BN heater, which is in the direct contact with the back side of the crystal. The temperature is measured by an N-type thermocouple placed between the heater and the sample's back side. In the high-pressure regime, high flow of room-temperature gas supplied to the surface cools the surface down by a certain amount, which should be accounted for. To do this, we have performed a number of calibration tests, for which purpose a dummy iron sample with two thermocouples spot-welded to both front and back sides was subjected to a series of temperature measurements at various gas flow and pressure conditions. The results of the tests are briefly summarized in Figure S4-1.

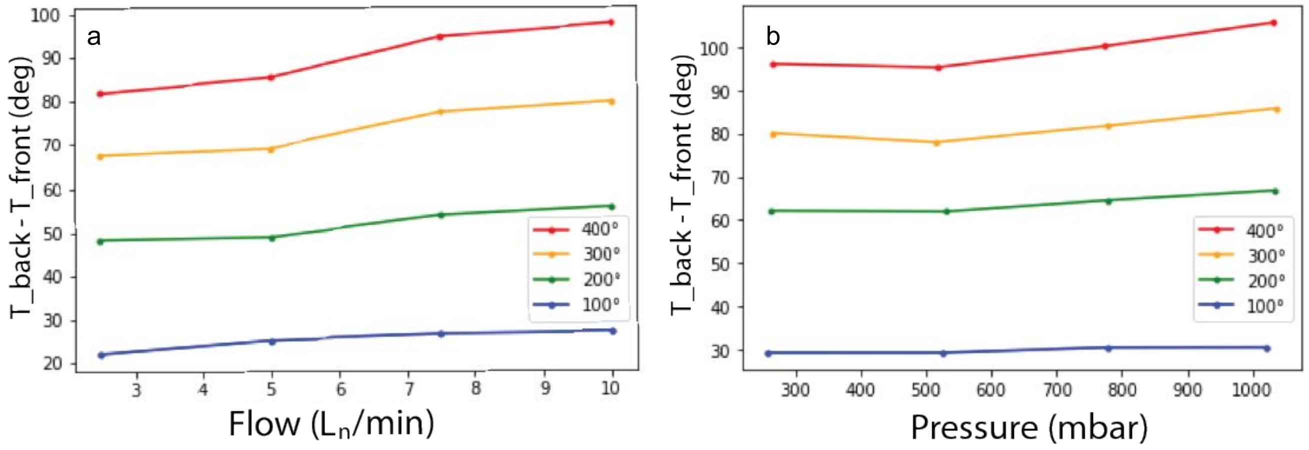

Figure S4-1. Calibration measurements of the temperature difference between the back and the front of the sample depending on the gas flow and pressure. In the panel (a), a constant pressure of 1 bar was maintained while changing the gas flow. In the panel (b), a constant flow of 10 L_n/min was maintained while changing the pressure. Each color correspond to the sample's backside temperature as indicated in the figure legend.

From the figure it can be seen that the main contribution to the surface temperature offset is related to the absolute temperature of the sample's back side, while the dependence on the pressure and the gas flow is rather small. Specifically, at flows below 3 L_n/min and pressures below 700 mbar (the upper limits in the currently discussed experiment), the temperature change due to these factors are negligible. Figure S4-2 shows the values of surface temperature offset measured at 100, 200, 300 and 400ºC (=sample backside temperature) and the non-linear interpolation of the measured points with 2nd and 3rd order polynomic functions. Both non-linear approximations give very similar values in the region of interest of the experiment, namely [100ºC; 400ºC]. Subtracting the corresponding values of the surface temperature offset from the thermocouple measurements allows for the determination of the actual temperature on the sample surface in the high-pressure regime.

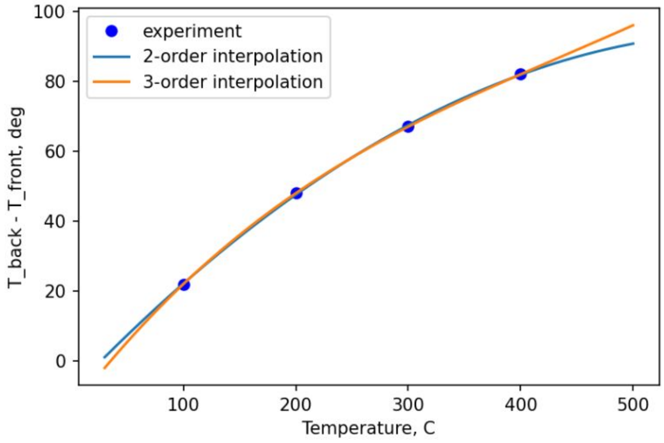

Figure S4-2. Measured values (as derived from the data shown in Figure S3-1) and non-linear interpolation of the surface temperature offset at pressures below 700 mbar and flows below 3 L_n/min.

A similar calibration of the temperature measurement was done for the diffraction experiment.

S5. Fermi level correction

The binding energy scale in all spectra was referenced to the Fermi level measured after every change of experimental conditions and stayed constant throughout the entire experiment. An example of recorded Fermi level and its fitting is shown in Figure S5-1.

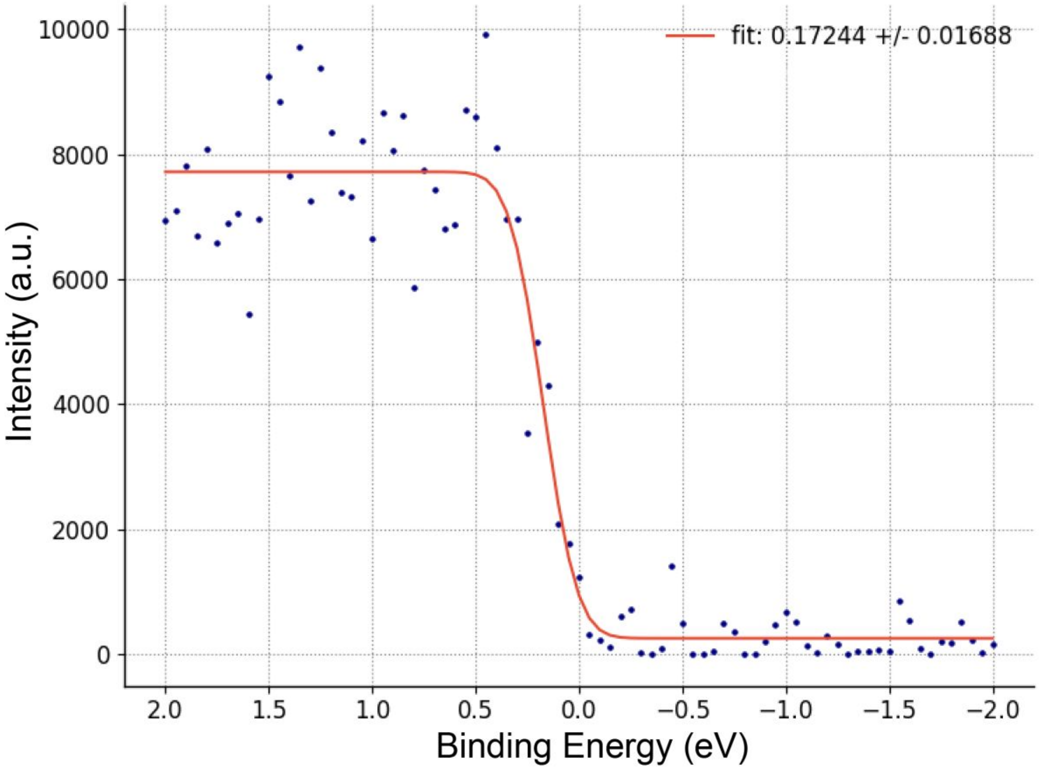

Figure S5-1. Fermi level recorded using 4.6 keV photons in vacuum at room temperature for Fe(110) single crystal surface (blue dots) and its fit (red line). Other experimental parameters are: Pass Energy = 100 eV; Analyzer Slit = 0.8 mm, curved; Energy Step = 50meV.

S6. XPS experimental data examples

Here we provide more examples of the experimental data for the reader's reference. Two representative sets of XPS measurements of a Fe(110) surface at 85 mbar and 550 mbar pressure and 1:1, 1:2, 1:4, and 1:10 CO to H_2 gas mixtures with 2-2.5 L_n/min total flow have been chosen for display in Figures S6-1 and S6-2. The data are normalized by the number of sweeps in every scan, by the dwell time, and by the background level on the low binding energy side of C 1*s* region (for scans of the same set taken simultaneously at the same conditions). To simplify the visual comparison of spectra, the intensity was also corrected by the corresponding values of the photoionization cross-section and the total scattering loss of photoelectrons in gas. In Figure S6-3, the C 1*s* level of the same measurements is shown in a different representation for more details and easier direct comparison. Representative mass-spectrometry data corresponding to the measurements at 550 mbar for 1CO : $1H_2$ and 1CO : $10H_2$ gas mixtures are also presented in Figures S6-4 – S6-7. Note that the MS signal m/z = 18, which corresponds to water, is greatly affected by interactions with the chamber walls of the differential pumping stage, which results in an accumulation in the system over time, and this signal can thus not be directly correlated to the activity at the indicated temperature.

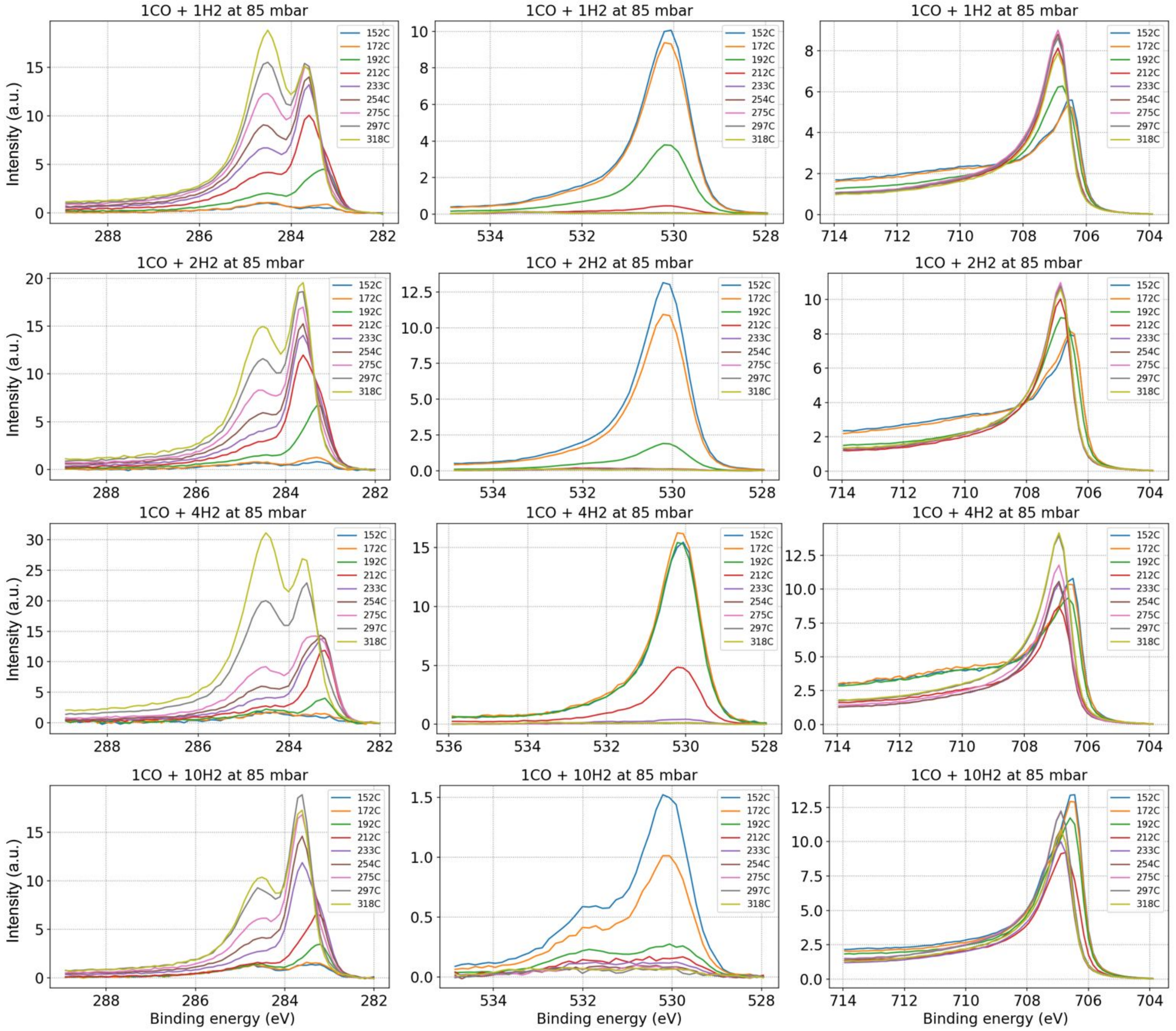

Figure S6-1. Photoelectron spectra recorded using 4.6 keV photons for C 1*s*, O 1*s*, and Fe 2$p_{3/2}$ regions at 85 mbar pressure and 1:1, 1:2, 1:4, and 1:10 CO to H_2 gas mixtures with 2-2.5 L_n/min total flow. Other experimental parameters are: Pass Energy = 100 eV; Analyzer Slit = 0.8 mm, curved; Energy Step = 50meV; Photon Energy = 4600 eV.

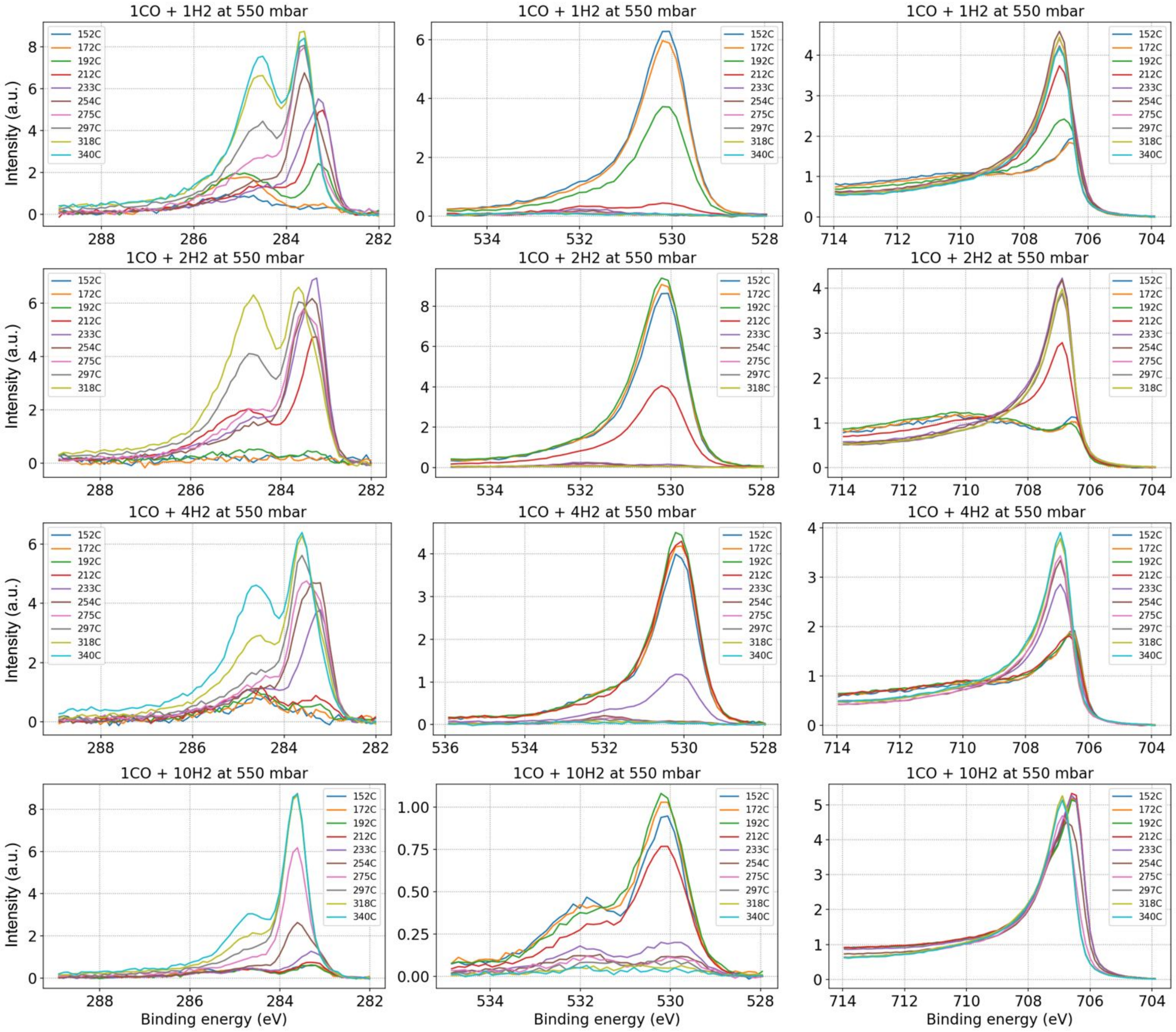

Figure S6-2. Photoelectron spectra recorded using 4.6 keV photons for C 1*s*, O 1*s*, and Fe 2$p_{3/2}$ regions at 550 mbar pressure and 1:1, 1:2, 1:4, and 1:10 CO to H_2 gas mixtures with 2-2.5 L_n/min total flow. Other experimental parameters are: Pass Energy = 100 eV; Analyzer Slit = 0.8 mm, curved; Energy Step = 50meV; Photon Energy = 4600 eV.

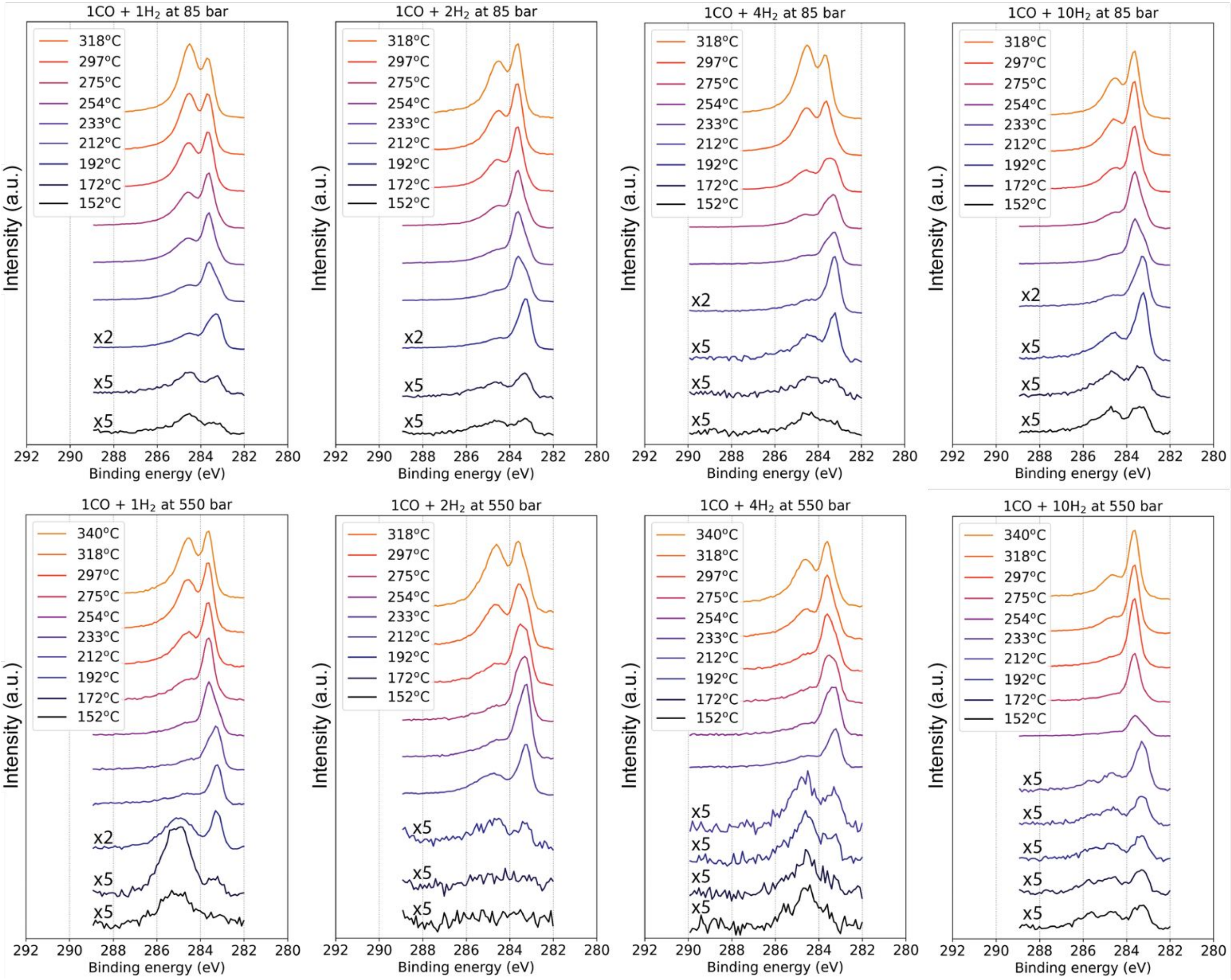

Figure S6-3. Photoelectron spectra recorded using 4.6 keV photons for C 1*s* region in 1:1, 1:2, 1:4, and 1:10 CO to H_2 reaction gas mixtures at total flows between 2 and 2.5 L_n/min and pressures of 85 mbar (top row) and 550 mbar (bottom row). Note that selected lower-temperature spectra are magnified by the factors of two and five for better visibility of fine details. Other experimental parameters are: Pass Energy = 100 eV; Analyzer Slit = 0.8 mm, curved; Energy Step = 50meV; Photon Energy = 4600 eV.

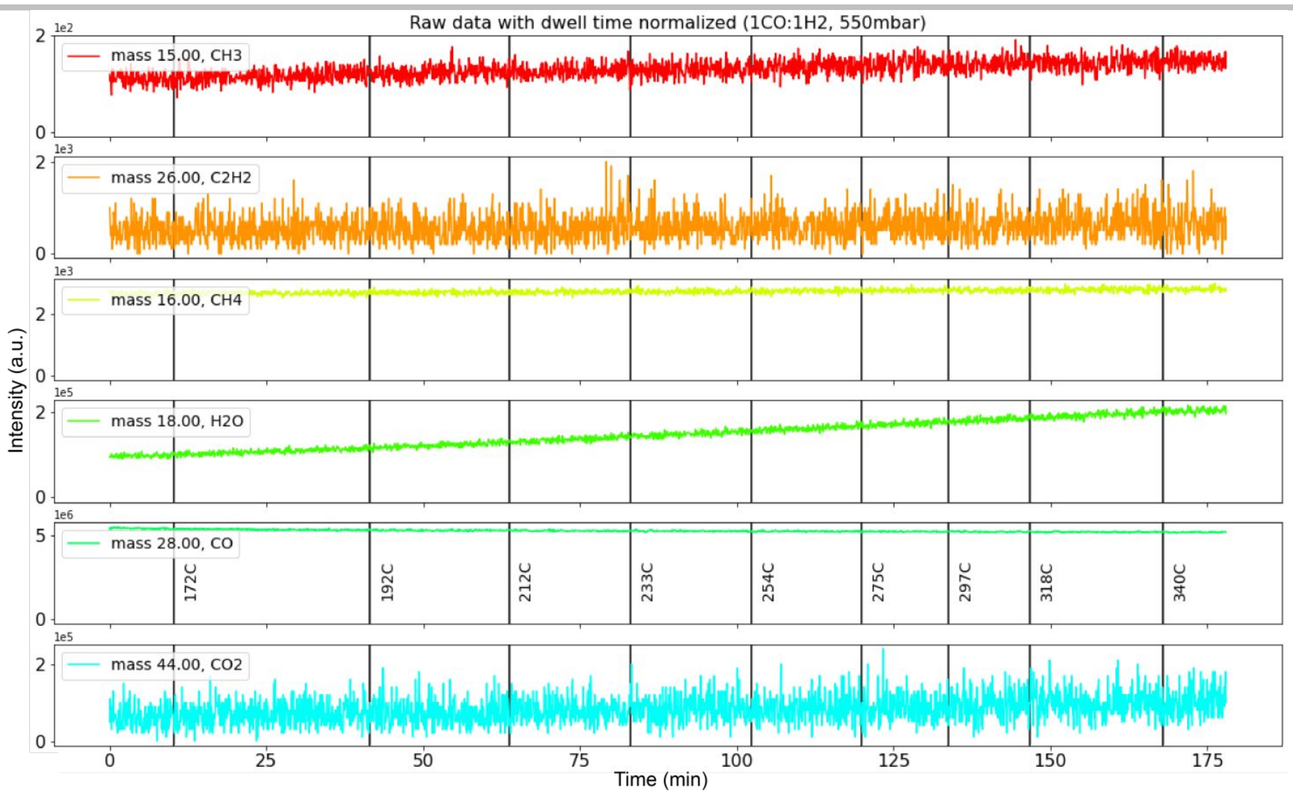

Figure S6-4. Mass-spectrometry signal corresponding to the set of measurements recorded at 550 mbar in the 1CO:1H$_2$ gas mixture at 2 L$_n$/min total flow. The data are normalized only by the dwell time. The moments of temperature increase are indicated in the CO signal panel.

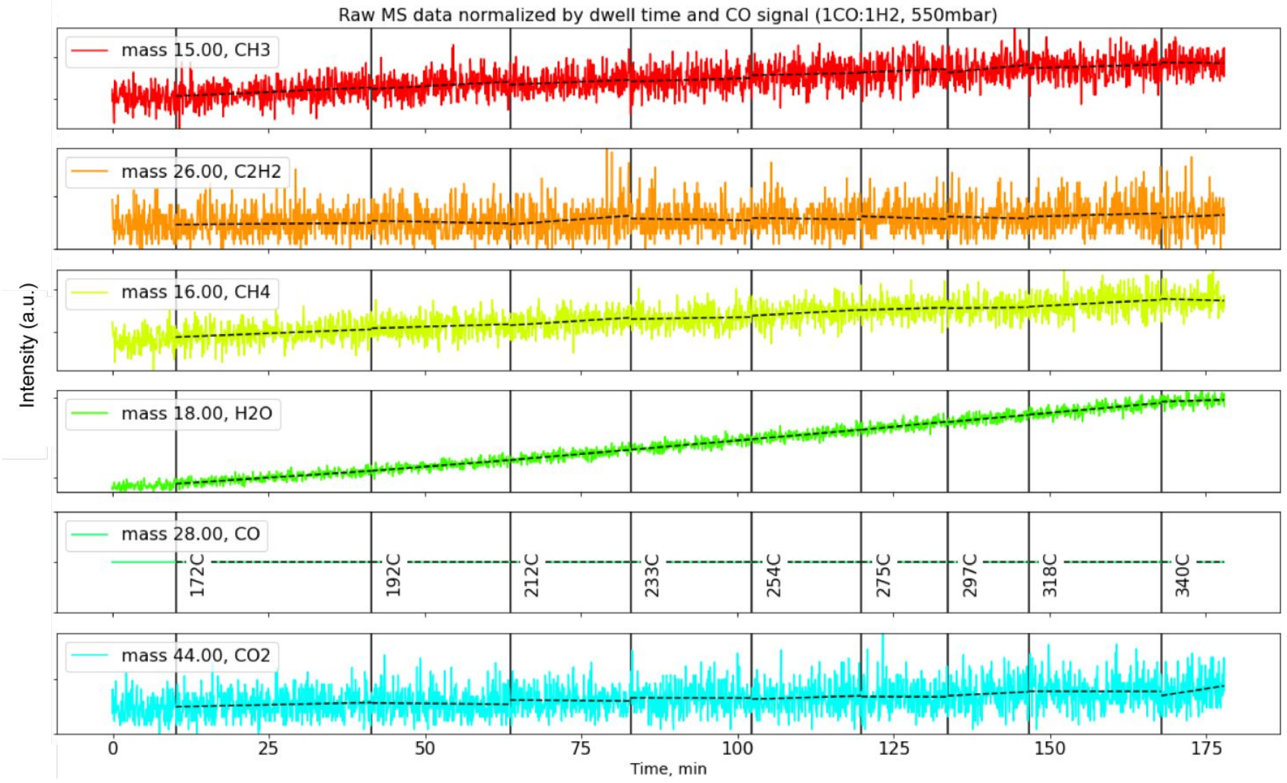

Figure S6-5. Mass-spectrometry signal corresponding to the set of measurements recorded at 550 mbar in the 1CO:1H$_2$ gas mixture at 2 L$_n$/min total flow. The data are normalized by the dwell time and CO signal. Broken line shows linear fits of every temperature interval. The moments of temperature increase are indicated in the CO signal panel.

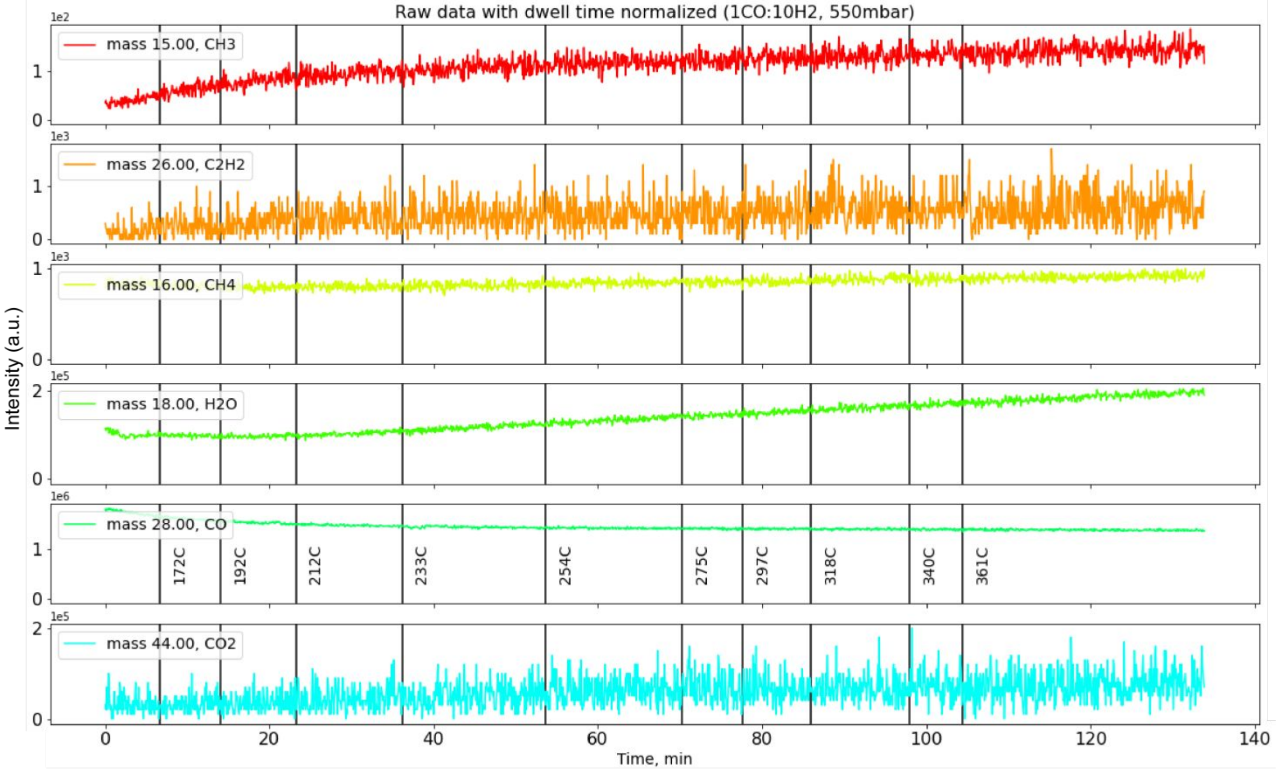

Figure S6-6. Mass-spectrometry signal corresponding to the set of measurements recorded at 550 mbar in the 1CO:10H_2 gas mixture at 2.5 L_n/min total flow. The data are normalized by the dwell time. The moments of temperature increase are indicated in the CO signal panel.

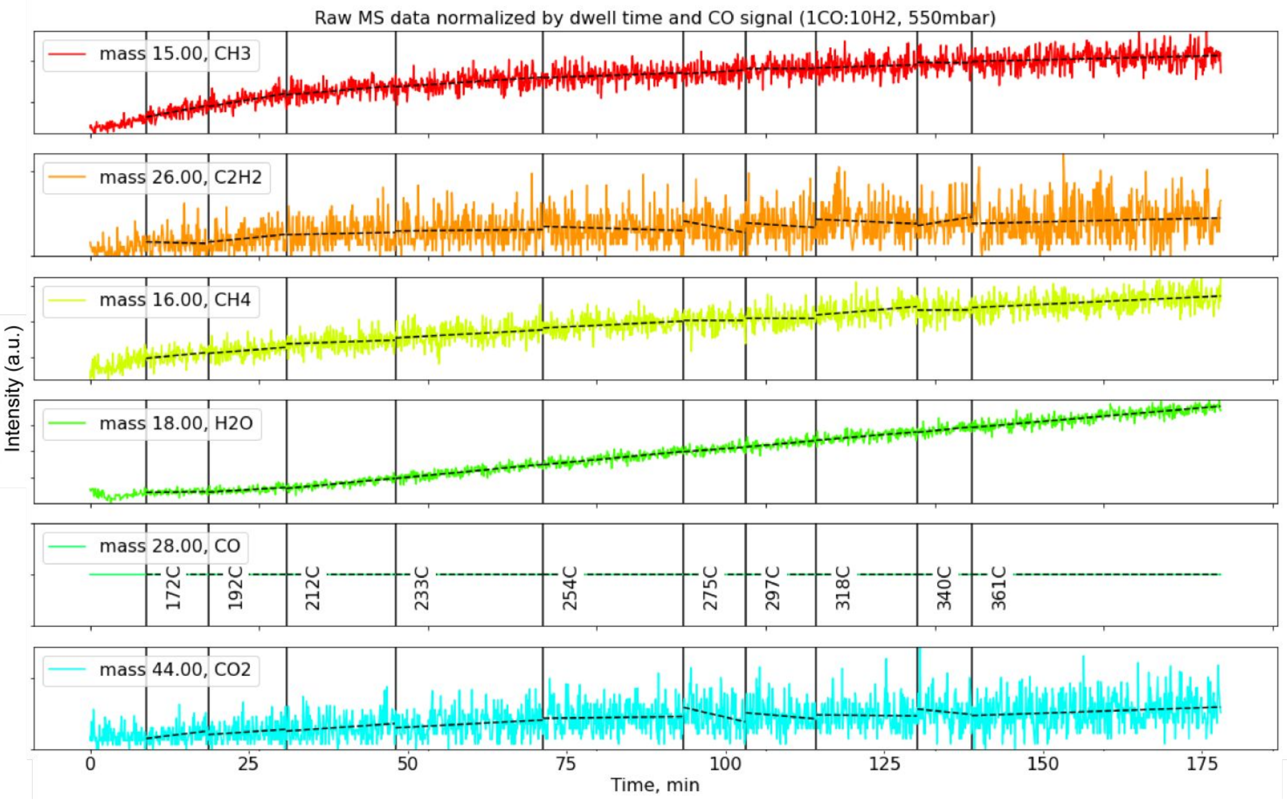

Figure S6-7. Mass-spectrometry signal corresponding to the set of measurements recorded at 550 mbar in the 1CO:10H_2 gas mixture at 2.5 L_n/min total flow. The data are normalized by the dwell time and CO signal. Broken line shows linear fits of every temperature interval. The moments of temperature increase are indicated in the CO signal panel.

S7. Diffraction experimental data examples

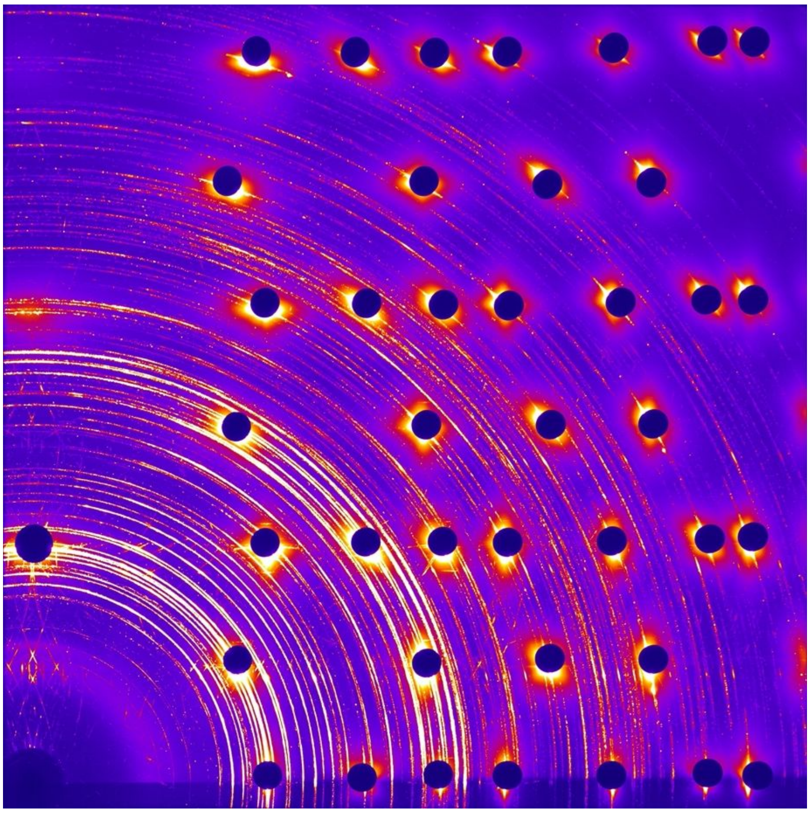

Figure S7-1. 2D diffraction pattern recorded using 83 keV photons for a Θ-Fe_3C covered surface at 350ºC after a set of measurements in $1CO{:}1H_2$ reaction gas mixture at 150 mbar total pressure.

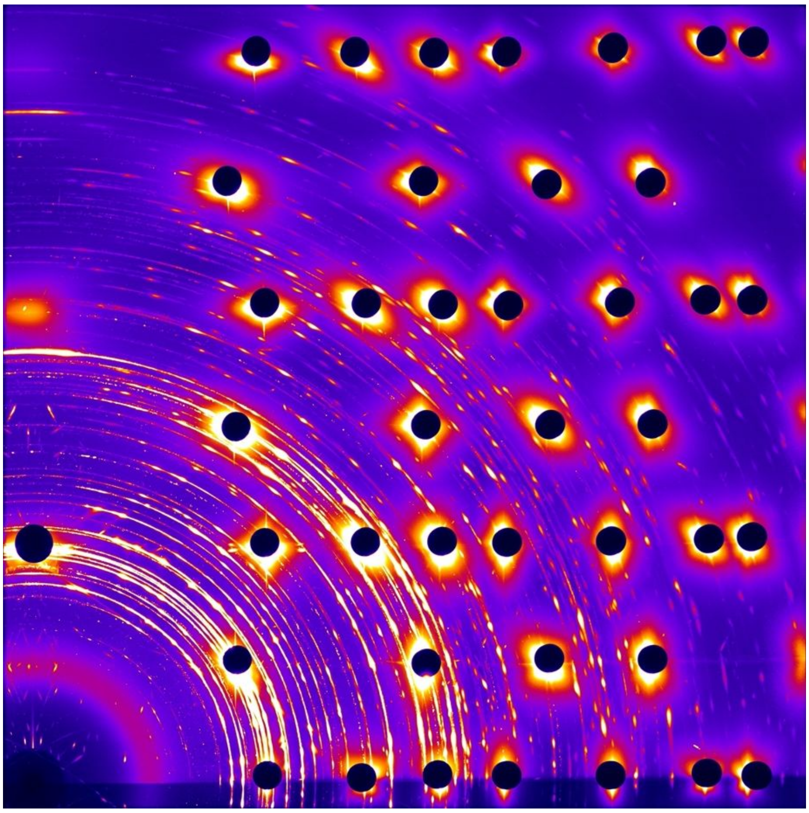

Figure S7-2. 2D diffraction pattern recorded using 83 keV photons for a Θ-Fe_3C covered surface at 350ºC after a set of measurements in $1CO{:}4H_2$ reaction gas mixture at 150 mbar total pressure.

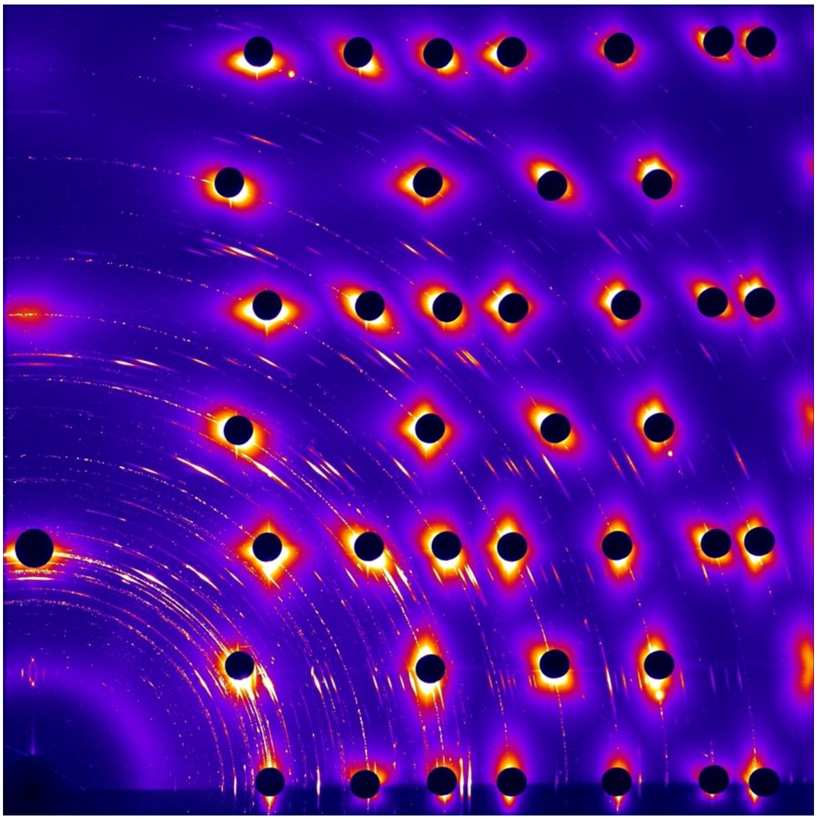

Figure S7-3. 2D diffraction pattern recorded using 83 keV photons for a Θ-Fe_3C covered surface at 350ºC after a set of measurements in 1CO:10H_2 reaction gas mixture at 150 mbar total pressure.

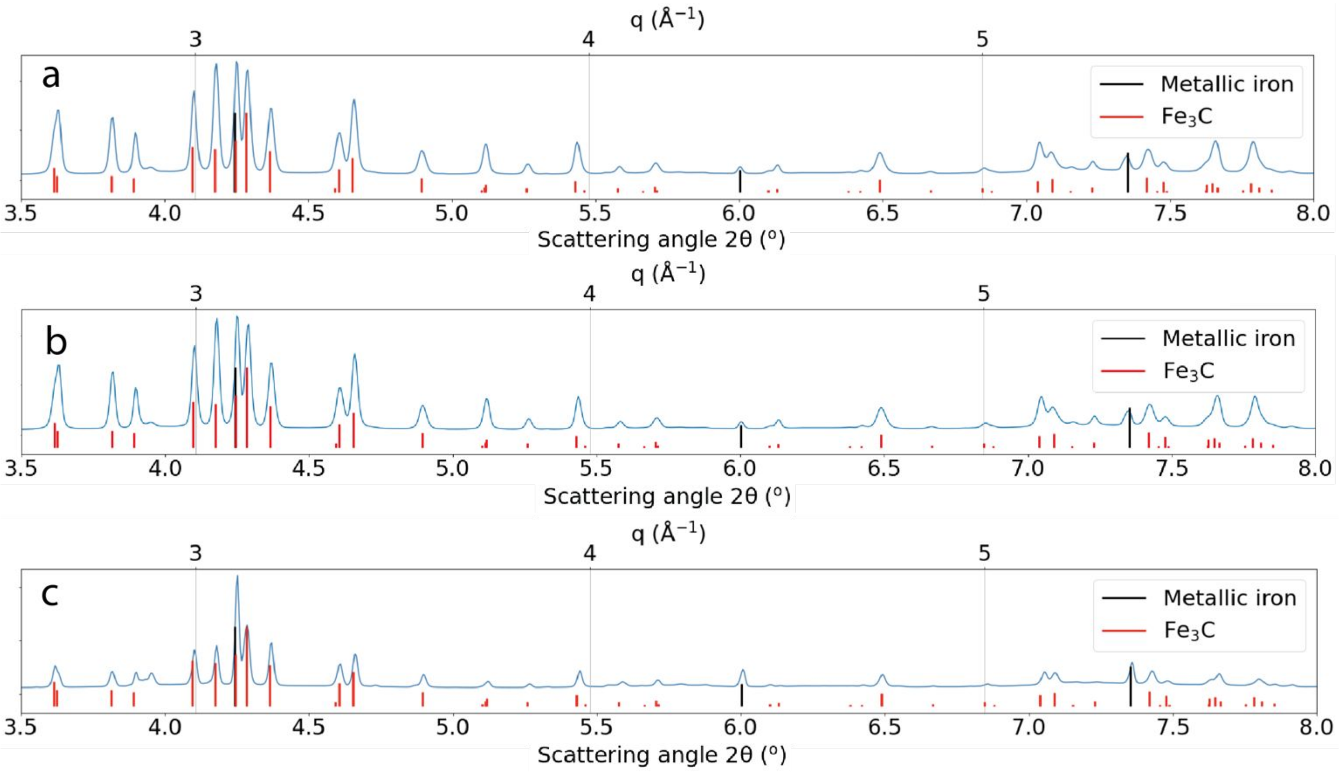

Figure S7-4. A circularly integrated representation of the 2D pattern in figures (a) S7-1, (b) S7-2, and (c) S7-3 with indicated reference values for metallic iron (black lines) and Θ-Fe_3C (red lines).

S8. Example of VASP INCAR file for calculating 1s and 2p binding energy

1s

```
INCAR created by Atomic Simulation Environment
 ENCUT = 520.000000
 SIGMA = 0.100000
 CLZ = 1.000000
 EDIFF = 1.00e-06
 ALGO = Normal
 GGA = PE
 PREC = Accurate
 IBRION = -1
 ISMEAR = -1
 ISPIN = 2
 ISTART = 1
 ISYM = 0
 KPAR = 8
 LORBIT = 10
 NBANDS = 1662
 NELM = 2000
 NPAR = 16
 NSIM = 8
 NSW = 0
 NWRITE = 2
 ICORELEVEL = 2
 CLNT = 1
 CLN = 1
 CLL = 0
 NEDOS = 1200
 LCHARG = .TRUE.
 LPLANE = .TRUE.
 LWAVE = .TRUE.
 LREAL = Auto
```

2p

```
INCAR created by Atomic Simulation Environment
 ENCUT = 520.000000
 SIGMA = 0.100000
 CLZ = 1.000000
 EDIFF = 1.00e-06
 ALGO = Normal
 GGA = PE
 PREC = Accurate
 IBRION = -1
 ISMEAR = -1
 ISPIN = 2
 ISTART = 1
 ISYM = 0
 KPAR = 8
 LORBIT = 10
 NBANDS = 1662
 NELM = 2000
 NPAR = 16
 NSIM = 8
 NSW = 0
 NWRITE = 2
 ICORELEVEL = 2
 CLNT = 1
 CLN = 1
```

```
CLL = 0
NEDOS = 1200
LCHARG = .TRUE.
LPLANE = .TRUE.
LWAVE = .TRUE.
LREAL = Auto
```

References

(1) Amann, P.; Degerman, D.; Lee, M. T.; Alexander, J. D.; Shipilin, M.; Wang, H. Y.; Cavalca, F.; Weston, M.; Gladh, J.; Blom, M.; Björkhage, M.; Löfgren, P.; Schlueter, C.; Loemker, P.; Ederer, K.; Drube, W.; Noei, H.; Zehetner, J.; Wentzel, H.; Åhlund, J.; Nilsson, A. A High-Pressure x-Ray Photoelectron Spectroscopy Instrument for Studies of Industrially Relevant Catalytic Reactions at Pressures of Several Bars. *Rev. Sci. Instrum.* **2019**, *90* (10).

(2) Schlueter, C.; Gloskovskii, A.; Ederer, K.; Piec, S.; Sing, M.; Claessen, R.; Wiemann, C.; Schneider, C. M.; Medjanik, K.; Schönhense, G.; Amann, P.; Nilsson, A.; Drube, W. New HAXPES Applications at PETRA III. *Syncrotron Radiat. News* **2018**, *4* (31), 29–35.

(3) Schlueter, C.; Gloskovskii, A.; Ederer, K.; Schostak, I.; Piec, S.; Sarkar, I.; Matveyev, Y.; Lömker, P.; Sing, M.; Claessen, R.; Wiemann, C.; Schneider, C. M.; Medjanik, K.; Schönhense, G.; Amann, P.; Nilsson, A.; Drube, W. The New Dedicated HAXPES Beamline P22 At. In *AIP Conference Proceedings*; 2019; Vol. 2054, p 040010.

(4) Gustafson, J.; Shipilin, M.; Zhang, C.; Stierle, A.; Hejral, U.; Ruett, U.; Gutowski, O.; Carlsson, P.; Skoglundh, M.; Lundgren, E. High-Energy Surface X-Ray Diffraction for Fast Surface Structure Determination. *Science* **2014**, *343* (6172), 758–761.

(5) Shipilin, M.; Hejral, U.; Lundgren, E.; Merte, L. R.; Zhang, C.; Stierle, A.; Ruett, U.; Gutowski, O.; Skoglundh, M.; Carlsson, P.-A.; others. Quantitative Surface Structure Determination Using in Situ High-Energy SXRD: Surface Oxide Formation on Pd(100) during Catalytic CO Oxidation. *Surf. Sci.* **2014**, *630*, 229–235.

(6) van Rijn, R.; Ackermann, M. D.; Balmes, O.; Dufrane, T.; Geluk, A.; Gonzalez, H.; Isern, H.; de Kuyper, E.; Petit, L.; Sole, V. A.; Wermeille, D.; Felici, R.; Frenken, J. W. M. Ultrahigh Vacuum/High-Pressure Flow Reactor for Surface x-Ray Diffraction and Grazing Incidence Small Angle x-Ray Scattering Studies Close to Conditions for Industrial Catalysis. *Rev. Sci. Instrum.* **2010**, *81* (1), 014101.

(7) Takata, Y.; Kayanuma, Y.; Yabashi, M.; Tamasaku, K.; Nishino, Y.; Miwa, D.; Harada, Y.; Horiba, K.; Shin, S.; Tanaka, S.; Ikenaga, E.; Kobayashi, K.; Senba, Y.; Ohashi, H.; Ishikawa, T. Recoil Effects of Photoelectrons in a Solid. *Phys. Rev. B - Condens. Matter Mater. Phys.* **2007**, *75* (23), 1–4.
